# A Chemically Defined Culture for Tooth Reconstitution

**DOI:** 10.1002/advs.202404345

**Published:** 2024-11-27

**Authors:** Ziwei Zhang, Hong Hu, Zhiheng Xu, Ce Shan, Hanyi Chen, Kun Xie, Kun Wang, Yifu Wang, Qing Zhu, Yike Yin, Haoyang Cai, Yunqiu Zhang, Zhonghan Li

**Affiliations:** ^1^ Center of Growth Metabolism and Aging Key Laboratory of Bio‐Resource and Eco‐Environment of Ministry of Education Animal Disease Prevention and Food Safety Key Laboratory of Sichuan Province College of Life Sciences Sichuan University 24 South Section 1, 1st Ring Road Chengdu 610065 China; ^2^ Department of Anesthesiology West China Second University Hospital, Key Laboratory of Birth Defects and Related Diseases of Women and Children of Ministry of Education Sichuan University No. 20, Section 3, South Renmin Road Chengdu 610041 China; ^3^ State Key Laboratory of Oral Disease West China Hospital of Stomatology Sichuan University No. 14, Section 3, South Renmin Road Chengdu 610041 China; ^4^ Yunnan Key Laboratory of Stomatology Department of Pediatric Dentistry The Affiliated Stomatology Hospital of Kunming Medical University Kunming Medical University No. 1088, Mid‐Haiyuan Road Kunming 650500 China

**Keywords:** enamel induction, serum‐free, tooth reconstitution, toothoid

## Abstract

It is known for decades that dental epithelium and mesenchyme can reconstitute and regenerate a functional tooth. However, the mechanism of tooth reconstitution remains largely unknown due to the lack of an efficient in vitro model. Here, a chemically defined culture system is established that supports tooth reconstitution, further development with normal anatomy, and prompt response to chemical interference in key developmental signaling pathways, termed as toothoids. By using such a system, it is discovered that, during reconstitution, instead of resetting the developmental clock, dental cells reorganized and restarted from the respective developmental stage where they are originally isolated. Moreover, co‐stimulation of Activin A and Hedgehog/Smoothened agonist (SAG) sustained the initial induction of tooth fate from the first branchial arch, which would be otherwise quickly lost in culture. Furthermore, activation of Bone Morphogenetic Protein (BMP) signaling triggered efficient enamel formation in the late‐stage toothoids, without affecting the normal development of ameloblasts. Together, these data highlight the toothoid culture as a powerful tool to dissect the molecular mechanisms of tooth reconstitution and regeneration.

## Graphical abstract

A chemically defined culture is established for tooth reconstitution, where the reconstituted tooth organoids could continuously develop and generate multiple dental lineages. Using such culture, tooth reconstitution is found not to reset the developmental clock, dental fate commitment from the first branchial arch requires co‐stimulation of Activin A and SAG, and enamel formation by ameloblasts requires activation of BMP signaling.

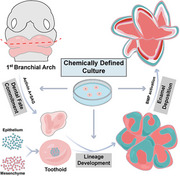

## 1 Introduction

Teeth and bones are the major mineralized organs/tissues in the human body. While bones are responsible for load‐bearing and locomotion, teeth play a critical role in our everyday life as part of our digestion system for food crushing and pulverization, providing support for facial tissues, and assistance in pronunciation during speech.^[^ ^1^ ^]^ The organogenesis of teeth relies on the interactive induction between epithelium and mesenchyme,^[^ ^2^ ^]^ one of the most conserved mechanisms also involved in developing many other mammalian organs such as the kidney,^[^ ^3^ ^]^ liver,^[^ ^4^ ^]^ lung,^[^ ^5^ ^]^ and hair follicles.^[^ ^6^ ^]^ It has been known for decades that embryonic dental epithelium and mesenchyme could reconstitute and regenerate a functional tooth^[^ ^7^ ^]^ and bioengineering of such tooth germs enabled total organ replacement in situ,^[^ ^8^ ^]^ however, until today, the molecular mechanisms governing the reconstitution process remains elusive, especially why cells at complete different developmental stages could still retain the reconstitution capability and whether the reconstitution process itself would reset the developmental clock.

Classical studies on the developmental process in tooth morphogenesis relied heavily on animal models^[^ ^2^ ^]^ as well as genetic mutations detected in human patients,^[^ ^9^ ^]^ which have greatly deepened our understanding of the roles of key signaling pathways such as Fibroblast Growth Factors (FGFs),^[^ ^10^ ^]^ BMPs,^[^ ^11^ ^]^ Sonic Hedgehog (SHHs),^[^ ^12^ ^]^ and Wnts^[^ ^2^, ^13^ ^]^ as well as important component genes in regulating various aspects of tooth morphogenesis and pathologies. However, as tooth development was usually accomplished at embryonic stages, except for the dental root and eruption, genetic manipulations of key signaling genes often led to abnormalities in extended craniofacial regions, or even whole organisms, causing complications such as embryonic lethality and thus preventing further exploration of the molecular mechanisms driving dental epithelial‐mesenchymal interaction and tooth reconstitution.^[^ ^2^, ^11^ ^]^ Recent advances in single‐cell sequencing and spatial profiling started to reveal the complexity and functional diversity of dental cell compositions during development^[^ ^14^ ^]^ and in mature teeth.^[^ ^15^ ^]^ However, the enthusiasm was still dampened by the lack of an efficient in vitro culture system to recapitulate tooth development faithfully and to dissect cellular and molecular mechanisms driving the formation of different dental lineages during tooth reconstitution.

Recently, several groups reported the establishment of dental organoid cultures, especially for dental epithelium,^[^ ^14^, ^16^ ^]^ which would eventually differentiate into ameloblasts governing enamel formation. The organoid‐cultured dental epithelial cells were shown to express critical enamel matrix protein components, such as amelogenin (AMGN) and ameloblastin (AMBN), however, there was a lack of evidence for efficient and complete enamel formation as it typically requires complicated protein‐guided deposition of enamel rods (hydroxyapatite crystallites),^[^ ^11^, ^17^ ^]^ a process of which the mechanism remains unclear. For whole tooth reconstitution and regeneration, the current strategy remains unimproved from the originally reported usage of fetal bovine serum (FBS),^[^ ^8^, ^18^ ^]^ which contains hundreds of ill‐defined growth factors and metabolites^[^ ^19^ ^]^ and thus makes it a challenge to elucidate key factors that govern the tooth reconstitution.

Here, we reported the establishment of a chemically defined culture system for tooth reconstitution, which not only supported toothoid formation across multiple embryonic stages, but also promoted sequential induction of stage‐specific markers, formation of critical dental lineages including odontoblasts and ameloblasts, and prompt responses to chemical interference of development‐relevant signaling pathways. By using such a system, we further discovered that tooth reconstitution did not involve resetting the developmental clock, but instead restarted from the stages where the cells were originally isolated. Moreover, sustained stimulation with Activin A and SHH activator was required to maintain the tooth potential from the initiation stage of the first branchial arch. Furthermore, activation of BMP signaling was identified as the trigger to stimulate efficient enamel secretion and maturation in the toothoids, without affecting overall lineage development of ameloblasts. Blocking BMP signaling strongly inhibited enamel induction, while activating it promoted enamel formation even in otherwise non‐permissive conditions. Together, these data highlight the toothoid culture system as a powerful tool to dissect key principles of tooth reconstitution and may guide the future development of tooth regeneration strategies.

## 2 Results

### 2.1 Developing a Chemically Defined Culture for Tooth Reconstitution

To track the development of the reconstituted tooth germs, a dual‐fluorescence reporter mouse model we constructed previously was used,^[^ ^20^ ^]^ where expression of epithelial *Pitx2* and mesenchymal *Msx1* could be monitored through copGFP and tdTomato expression respectively across different developmental stages (Figure S1A, Supporting Information). When the reconstituted tooth germs from E14.5 cells were cultured in the traditional FBS‐containing medium (Table S1A, Supporting Information), dental epithelium, and mesenchyme would spontaneously reorganize and form multiple epithelial foci surrounded by dental mesenchymal cells, which then continued to develop into cap stage‐like toothoids within 10 days of culture (Figure S1B, Supporting Information). When these toothoids were transplanted under the renal capsule for 2 weeks, individual tooth‐like structures with enamel, dentin/pre‐dentin, odontoblasts, and dental papilla regions could be readily observed (Figure S1C, Supporting Information). The mineralization of developed tooth‐like structures was also confirmed by Micro‐computed tomography (Micro‐CT) analysis (Figure S1D, Supporting Information).

**Figure 1:**
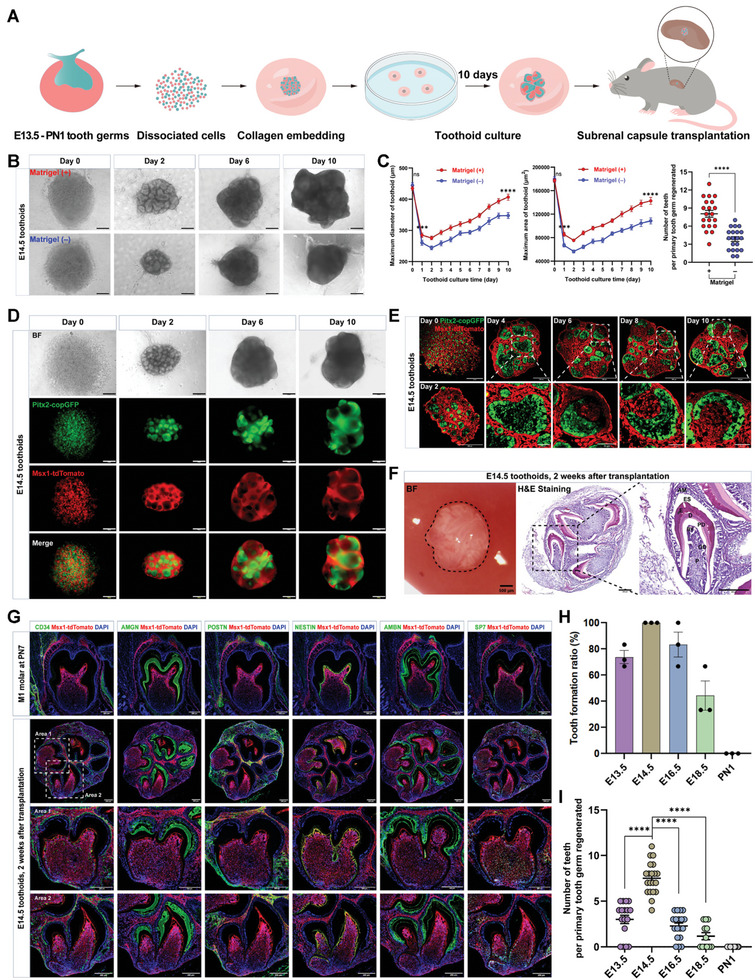
A chemically defined culture supported tooth reconstitution and regeneration. A) Schematic of the tooth germ reconstitution and toothoid formation by using the dual‐color mouse model. Tooth germs at E13.5‐PN1 were isolated from homozygous dual‐color mice (Pitx2:P2A‐GFP; Msx1:P2A‐tdTomato) and primary cells were aggregated and embedded with collagen. The reconstituted tooth germs were cultured for 10 days in vitro before kidney capsule transplantation. B) Representative time‐course images of toothoids cultured with/without Matrigel. Scale bars: 200 µm. C) Quantitative analysis indicated addition of 2% Matrigel in the defined medium strongly promoted toothoid growth. Daily measurements of the maximum area (left) and diameter (middle) were carried out. The number of teeth per toothoid (right) was quantified after 2 weeks of transplantation in vivo. Error bars represented data as mean ± SEM from three independent experiments, *n* = 20. Statistics: *p*‐values were calculated using two‐tailed unpaired Student's *t*‐test by SPSS v27. ***p < 0.001, ****p < 0.0001. ns: not significant. D) Representative time‐course images of dual‐color toothoids cultured in the chemically defined medium in vitro. The progressions of self‐reorganization, growth, and differentiation in toothoids were closely monitored by dual fluorescence imaging. Scale bars: 200 µm. E) Frozen sections and confocal analysis revealed the development processes of cultured toothoids. Typical morphology of the developing tooth germs could be observed in these toothoids. Samples were immunostained with copGFP and tdTomato antibodies. Scale bars: 200 µm and 50 µm (zoom‐in). F) The toothoids still retained the capability to develop into tooth‐like structures upon further transplantation in vivo. Multiple tooth‐like structures were observed in each toothoid by H&E staining after 2 weeks of transplantation. AM: ameloblasts; ES: enamel space; E: enamel; D: dentin; PD: pre‐dentin; OD: odontoblasts; P: dental pulp; BV: blood vessel. Scale bars: 500 µm (left) and 200 µm (middle and right). G) Tooth‐like structures developed from the transplanted toothoids displayed typical tooth anatomy. Samples were transplanted in vivo for 2 weeks before analysis. Markers used: Enamel matrix proteins (amelogenin, AMGN; ameloblastin, AMBN); Odontoblasts (NESTIN, SP7); Endothelial cells (CD34); Periodontal tissues (POSTN). Scale bars: 200 µm. H) The toothoids derived from embryonic stages, but not postnatal ones, retained the capability for tooth formation. Error bars represented data as mean ± SEM from three independent experiments. I) Toothoids (molar) from embryonic stages could generate tooth‐like structures. PN1 toothoids did not form any tooth‐like structures. Only toothoids with both crown and root would be quantified. Error bars represented data as mean ± SEM from three independent experiments: E13.5, *n* = 17; E14.5, *n* = 20; E16.5, *n* = 18; E18.5, *n* = 12; PN1, *n* = 12. Statistics: *p*‐values were calculated using one‐way ANOVA with Dunnett's test by SPSS v27. ****p < 0.0001.

Next, we sought to investigate if such developmental potential of toothoids could also be maintained when cultured in the chemically defined medium (**Figure** **1**A). We isolated primary cells from tooth germs at multiple developmental stages for tooth reconstitution (Figure S2A, Supporting Information). When dispersed epithelial and mesenchymal cells were reorganized in a chemically defined medium with 2% Matrigel as the only supplement (Table S1B, Supporting Information), they exhibited a surprisingly robust potential to reorganize into toothoids and continuously grow in the defined medium (Figure 1B,C; Figure S2B, Supporting Information). Dual color‐labeled toothoid culture further revealed the formation of multiple epithelial foci (copGFP^+^) surrounded by dental mesenchyme (tdTomato^+^) (Figure 1D). Immunostaining and confocal imaging revealed the formation of tooth germ‐like structures within these foci (Figure 1E). When transplanted under the kidney capsule, these toothoids further developed into tooth‐like structures with dental pulp, odontoblasts, pre‐dentin/dentin, and enamel tissues, except those reconstituted from the PN1 stage (Figure 1F; Figure S2C, Supporting Information). Notably, cells from E12.5 tooth germs could not self‐organize for reconstitution, but intact tooth germs would be able to continuously develop in the defined culture (Figure S2D, Supporting Information). Immunostaining of different dental markers such as AMGN and AMBN (markers for dental enamel organic matrix proteins),^[^ ^11^ ^]^ Nestin (a marker for odontoblasts),^[^ ^21^ ^]^ Periostin (POSTN) (a marker for periodontal ligament tissues) (PDL),^[^ ^22^ ^]^ CD34 (a marker of hematopoietic stem/progenitor cells and vascular endothelial cells),^[^ ^23^ ^]^ and Osterix (OSX or SP7, an osteoblast‐specific marker and highly expressed in odontoblasts from the late‐bell stage)^[^ ^24^ ^]^ further confirmed the tooth‐like structures had normal dental anatomy, similar to the M1 molar isolated at PN7 stage (Figure 1G). Quantitative analysis indicated that within such a culture system, toothoids could be reconstituted by dental cells from multiple embryonic stages, but the capability was lost postnatally and E14.5 cells could generate the highest number of tooth‐like structures per toothoid (Figure 1H,I).

In addition to molar teeth, we also test if such a culture system could be used for incisor reconstitution. Indeed, primary dental cells from the E14.5 incisor could also self‐reorganize and form epithelial foci surrounded by mesenchymal cells (Figure S3A,B, Supporting Information). Upon transplantation, the reconstituted incisor tooth germs could further develop into tooth‐like structures (Figure S3C, Supporting Information). Interestingly, compared with molar ones, the number of teeth per toothoid was much lower in incisor toothoids, while each tooth‐like structure was significantly larger (Figure S3D, Supporting Information), suggesting that the incisor and molar dental cells might have distinct reconstitution capabilities. In addition, we also isolated and reconstituted incisor tooth germs from multiple other stages (E13.5‐PN1), and most of them developed into tooth‐like structures, except for those at the E18.5 and later stages (Figure S3E–G, Supporting Information). Although the E16.5 and E17.5 incisor toothoids formed dentin, odontoblasts, and dental pulp structures, they lacked ameloblasts and enamel formation (Figure S3F,G, Supporting Information). These data indicated that the incisor development might progress faster than the molar at the same stage, resulting in an earlier loss of self‐organization potential.

Together, these data suggested that toothoids could be cultured in a minimum chemically defined medium and still retained the capability to develop into tooth‐like structures upon transplantation.

### 2.2 Recapitulating Dental Development Using Toothoids

**Figure 2:**
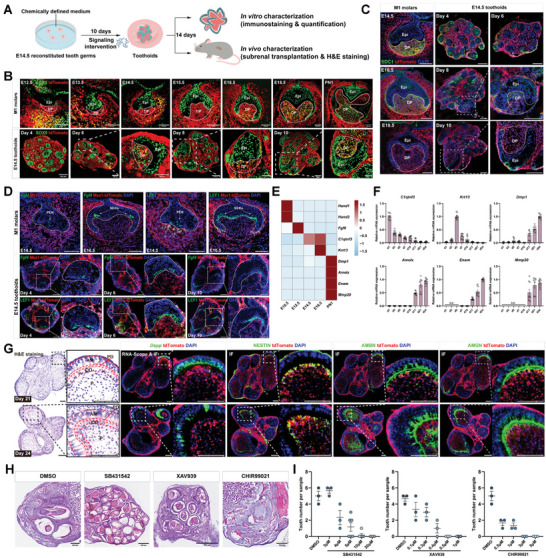
Recapitulating the tooth development using the chemically defined toothoid culture system. A) The experimental strategy for analyzing toothoid development. The E14.5 toothoids were continuously cultured in the chemically defined medium in vitro to analyze the derivation of dental lineage cells. Small molecules treated toothoids were transplanted in vivo before analysis. B,C) Direct comparison of dental markers (SOX9 & SDC1) revealed similarity between toothoids and M1 molars. DAPI was used for nuclear staining. Epi: epithelium; DP: dental papilla. Scale bars: 100 and 50 µm (zoom‐in) in B; 100 µm in (C). D) Formation of enamel knots in the toothoids. The presence of enamel knots was confirmed by RNAscope and immunostaining. Enamel knot marker: *Fgf4* and LEF1. Scale bars: 100 and 50 µm (zoom‐in). E) Heatmap for stage‐specific dental markers. F) Sequential induction of stage‐specific dental markers in toothoids. Toothoids were cultured in vitro for 24 days, and samples were collected every 2–3 days for RT‐qPCR analysis. Error bars represented data as mean ± SEM from three independent experiments with triplicates. G) Derivation of dental lineage cells in the toothoids. The formation of ameloblasts and odontoblasts in toothoids was confirmed by H&E staining, RNAscope, and immunostaining. AM: ameloblasts; PD: pre‐dentin; OD: odontoblasts; P: dental pulp; Enamel matrix proteins: AMGN, AMBN; Odontoblasts: NESTIN, SP7; dentin sialophosphoprotein: *Dspp*. Scale bars: 100 µm. H) Toothoids promptly responded to the small molecule‐mediated intervention of key signaling pathways. The toothoids were treated with 10 µm SB431542 (TGF‐β inhibitor), 0.5 µm XAV939 (Wnt inhibitor), or 3 µm CHIR99021 (Wnt activator) for 10 days before renal capsule transplantation for 2 weeks. DMSO was used as the solvent control. Scale bars: 200 µm. I) Quantitative analysis of the dose‐dependent responses of small molecules on toothoids. The toothoids were treated with various concentrations of SB4315421, XAV939, or CHIR99021 for 10 days before transplantation. Only toothoids with both crown and root would be quantified. Error bars represented data as mean ± SEM from independent experiments of each group: DMSO, *n* = 3; SB431542, *n* = 3 (3, 4, and 20 µm) and *n* = 6 (5 and 10 µm); XAV939, *n* = 3 (0.1, 0.3, 0.4, and 0.5 µm) and *n* = 5 (1 µm); CHIR99021, *n* = 3 (0.5, 1, 3, and 5 µm).

Next, we sought to explore if the toothoids could recapitulate the dental developmental process entirely in vitro. To do this, single cells from E14.5 tooth germs were reconstituted and continuously cultured in the defined medium and the resulting toothoids were characterized in vitro and in vivo, with the M1 molars at different developmental stages as the comparison (**Figure** **2**A). Msx1^+^Sox9^+^ cells, a mesenchymal progenitor group previously reported to make major contributions to the final tooth formation,^[^ ^20^ ^]^ were initially detected surrounding the developing molar and gradually invaded the papilla region and became the major cell composition at later stages in molar tooth germs (Figure 2B, top). In the E14.5 toothoids, spots of mesenchymal Msx1^+^Sox9^+^ cells could be observed throughout the tissues in the initial stage (around day 4), gradually concentrated in the papilla regions at day 6 and became the major cell composition from day 8 to 10 (Figure 2B, bottom). A similar phenotype could also be observed from E13.5 toothoids (Figure S4A, Supporting Information). Moreover, the evaluation of additional marker *Sdc1* confirmed that the dynamic expression pattern of dental marker genes could also be observed in the toothoids, similar to the native M1 molar. SDC1 was initially expressed in the dental papilla region as well as dental epithelium from E14.5 to E16.5 and disappeared from the papilla region around E17.5–E18.5 in vivo (Figure 2C, left), while in the toothoids, expression of SDC1 was observed around day 8 in the papilla region of the reconstituted tooth germ and disappeared around day 10 (Figure 2C, right).

The enamel knots, including the primary (PEK) and secondary enamel knots (SEKs), serve as the crucial signaling center to provide positional cues essential for tooth morphogenesis and guide the development of tooth cusps.^[^ ^25^ ^]^ To investigate if enamel knots were formed in the toothoids, the expression of two representative markers, *Fgf4* ^[^ ^26^ ^]^ and *Lef1*,^[^ ^27^ ^]^ was evaluated by RNAscope and immunostaining respectively (Figure 2D, top). Indeed, in E14.5 toothoids, the PEK could be observed as early as day 4 and persisted until day 10 (Figure 2D, bottom). No SEKs were observed, possibly due to the size limits of the self‐organized tooth germs, all of which showed a single cusp (Figure 1F; Figure S2C, Supporting Information).

To test whether the toothoids could continue to develop and generate more mature dental lineage cells, they were cultured further until day 24 before characterization. We first re‐analyzed previously reported scRNA‐seq datasets from developing molar tooth germs and identified dental markers that had stage‐specific expression, including *Hand1/2*, *Fgf8*, *C1qtnf3*, *Krt13*, *Dmp1, Amelx, Enam*, and *Mmp20* (Figure 2E). RT‐qPCR analysis of toothoids at different time points confirmed the robust sequential induction of those stage‐specific dental markers (Figure 2F). Immunostaining/RNAscope analysis of ameloblasts and odontoblasts markers, including AMGN, AMBN, *Dspp*, and NESTIN, further confirmed the induction of matured dental lineage cells in the toothoids (Figure 2G). A similar observation was also made by immunostaining with SP7, which had a wide initial distribution within the developing tooth germ but would concentrate on odontoblasts at later developmental stages (E18.5 and PN1) (Figure S4B, Supporting Information, top). Similar enrichment of SP7 in the odontoblasts could be detected in the E14.5 toothoids as well (Figure S4B, Supporting Information, bottom). We also confirmed that toothoids from multiple other stages could continuously develop and generate mature dental lineage cells, similar to E14.5 toothoids (Figure S4C, Supporting Information).

Since many signaling pathways, such as transforming growth factor‐β (TGF‐β) and Wnt, play critical roles in various aspects of tooth development, we next sought to investigate if the toothoids would also respond to the modulation of these signaling pathways using chemical tool compounds. Three tool compounds were chosen, including SB431542, a TGF‐β receptor Alk5 inhibitor,^[^ ^28^ ^]^ XAV939, a tankyrase inhibitor that targets Wnt/β‐catenin signaling,^[^ ^29^ ^]^ and CHIR99021, a GSK3‐β inhibitor that is commonly used as a Wnt activator.^[^ ^30^ ^]^ Alk5 was known to regulate tooth initiation and mandible patterning^[^ ^31^ ^]^ and blocking it with SB431542 showed a dose‐dependent decrease of tooth formation in the toothoids along with increased epidermalization (Figure 2H,I; Figure S4D–F, Supporting Information). Similarly, blocking Wnt with XAV939 inhibited tooth formation and increased epidermalization as revealed by H&E staining and Keratin14 immunostaining (Figure 2H,I; Figure S4D–F, Supporting Information), which was also consistent with the previous report that loss of Wnt signaling resulted in tooth developmental arrest at the bud stage.^[^ ^24^, ^32^ ^]^ Moreover, continuous activation of Wnt signaling with CHIR99021, on the other hand, induced the formation of multiple dentin‐pulp complex‐ and enamel‐like structures (Figure 2H,I; Figure S4D,E, Supporting Information), making it impossible to quantify individual teeth formed within the toothoids. Importantly, it was also consistent with previous results that activation of Wnt signaling led to supernumerary phenotype in both mouse and human individuals.^[^ ^33^ ^]^ Altogether, these results indicated that the toothoids in the defined culture could recapitulate normal tooth development and exhibit a prompt response to the chemical modulation of key signaling pathways.

### 2.3 Tooth Reconstitution did not Reset the Developmental Clock

After confirming the robustness of the toothoid culture, we next sought to investigate the mechanism underneath tooth reconstitution. Dental epithelium and mesenchyme were known to be able to reconstitute and form a tooth organ^[^ ^34^ ^]^ and our previous results indicated that this inductive epithelial‐mesenchymal interaction existed only in cells from the embryonic stages (at least for M1 molar).^[^ ^14b^ ^]^ From the previous attempt to induce non‐dental cells to acquire odontogenic lineages, expression of early dental markers was observed in the induced cells^[^ ^35^ ^]^ but whether the tooth reconstitution would totally reset the developmental clock remains unclear.

**Figure 3:**
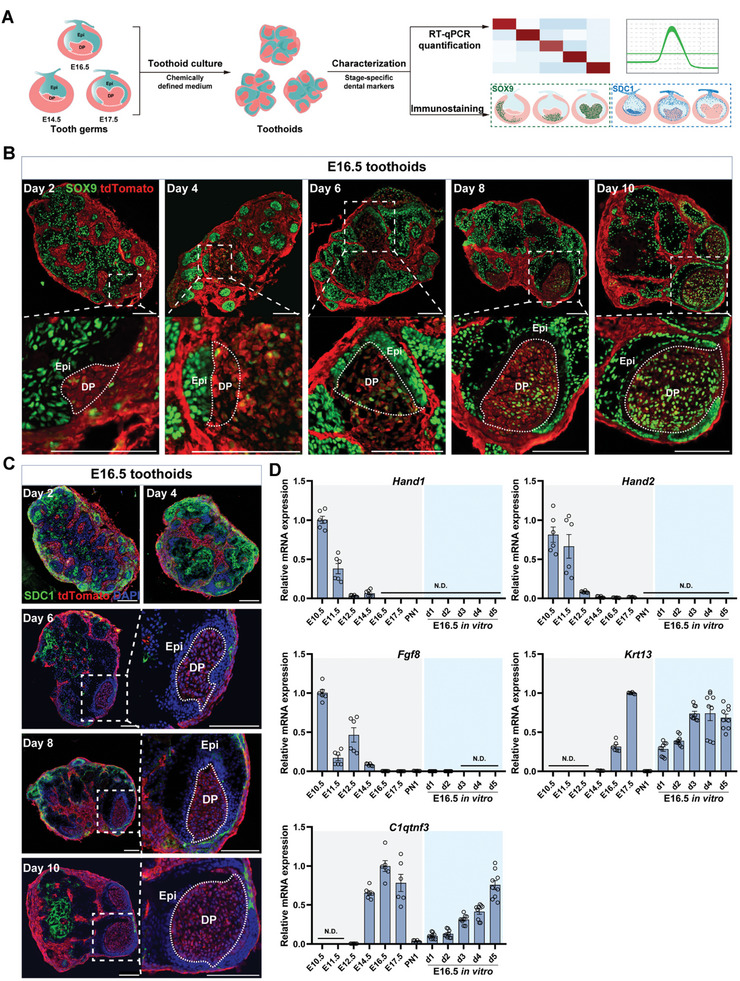
Tooth reconstitution did not reset the developmental clock but re‐initiated from the current stage. A) The experimental strategy to analyze tooth reconstitution using primary dental cells isolated from respective stages. The isolated tooth germs (E14.5, E16.5, and E17.5) were dissociated into single cells, reconstituted, and cultured in the chemically defined medium to form toothoids. Stage‐specific dental markers were then evaluated in these toothoids at different time points. B) Expression of SOX9 was already detected in the dental papilla region (dash line) as early as day 2 in the E16.5 toothoids culture. tdTomato was used to mark the mesenchymal cells. Scale bars: 100 µm. C) SDC1, a marker highly expressed in the dental papilla from E14.5–E16.5, was no longer detected in the E16.5 toothoids after day 6. Frozen sections were immunostained. DAPI was used for nuclear staining. Epi: epithelium; DP: dental papilla. Scale bars: 100 µm. D) Expression of stage‐specific dental markers indicated that the reconstituted toothoids did not restart from the initiation stage. The E16.5 toothoids were cultured in vitro for 5 days, and samples were collected every day for RNA extraction using the TRIzol method. Error bars represented data as mean ± SEM from at least two independent experiments with triplicates.

To investigate if reconstitution would reset the developmental clock for the dental cells, primary cells were isolated from tooth germs at E14.5, E16.5, and E17.5 for reconstitution. The purpose of the use of E16.5–E17.5 tooth germs was to provide an appropriate time window to track the expression of early stage‐specific markers if the reconstitution process indeed reset the developmental clock. The resulting toothoids were then characterized with dental stage‐specific markers identified from previous scRNA‐seq (**Figure** **3**A). SOX9 immunostaining revealed that Msx1^+^Sox9^+^ cells already appeared in the prospective dental papilla region at day 2, which was at least 2–4 days earlier than E14.5 toothoids (Figure 2B, bottom), and gradually became the major cell composition at day 10 (Figure 3B). This was not unexpected because Msx1^+^Sox9^+^ in the primary tooth germs already migrated into the papilla region at E16.5, while at E14.5 the same group of cells was still at the peripheral niche region (Figure 2B, top). However, it was surprising to find that SDC1 expression was never detected in the dental papilla region of the reconstituted E16.5 toothoids at day 6 and beyond (Figure 3C). If the reconstitution would reset the developmental clock, expression of SDC1 should be observed in the papilla region and then disappear at later stages, as indicated previously by E14.5 toothoids (Figure 2C).

This led to our hypothesis that reconstitution might not reset the clock but instead, the toothoids would resume the development from the respective stage they were initially isolated from. To test the hypothesis, expressions of stage‐specific dental markers were analyzed in the E16.5 toothoids harvested at different time points. *Hand1/2* and *Fgf8*, which were only expressed during the initial bud‐to‐cap stages, were never detected at any analyzed time points (Figure 3D). Meanwhile, *C1qtnf3* and *Krt13*, which were typically expressed from E14.5 to E17.5, were robustly induced in the reconstituted toothoids (Figure 3D). To rule out the possibility that it was specific to the E16.5 toothoids, those from two additional stages, E17.5 and E14.5, were also evaluated. In the E17.5 toothoids, we also observed the early appearance of Msx1^+^Sox9^+^ cells within the prospective papilla region and failed induction of SDC1 expression (Figure S5A,B, Supporting Information). RT‐qPCR analysis of stage‐specific dental markers revealed that not only early markers but also those normally induced at E16.5–E17.5 were not detected in the toothoids anymore (Figure S5C, Supporting Information). Similar observations could be made in the E14.5 toothoids as well, where markers expressed earlier than E14.5 were not induced at all in the toothoids (Figure S5D, Supporting Information). Together, these data highlighted a surprising finding that tooth reconstitution did not reset the developmental clock and the toothoids would restart from the developmental stages the cells were initially isolated from.

### 2.4 Tooth Induction from the First Branchial Arch

Although the minimum defined culture could support the development of toothoids or primary tooth germs from E12.5 and beyond, the isolated first branchial arch at E10.5 would quickly lose the capability to induce tooth formation in such conditions (Figure S6A, Supporting Information), suggesting that additional stimulation might be required. From previous studies in dental developmental biology,^[^ ^36^ ^]^ the mesenchyme‐derived Activin A acted as an early essential signal to initiate tooth development. Meanwhile, SHH^[^ ^37^ ^]^ and Wnt^[^ ^38^ ^]^ signaling were also required for tooth initiation from animal mutant analysis. Thus, we sought to evaluate if modulating these signaling pathways would help to maintain the tooth induction potential from the first branchial arch.

**Figure 4:**
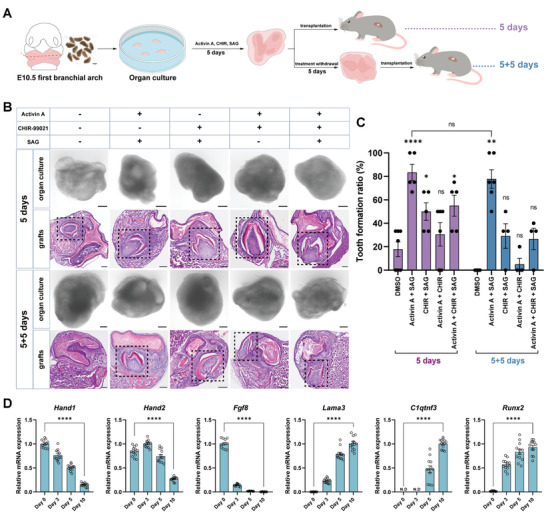
Activin A and SAG co‐stimulation maintained the tooth initiation potential of the first branchial arches. A) The experimental strategy. The rostral regions of E10.5 first branchial arches were cultured in the chemically defined medium and induced with small molecules and growth factors for 5 days, followed by either transplantation (5 days) or a 5‐day withdrawal of the inducing factors before transplantation (5 + 5 days). B) Co‐stimulation with Activin A and SAG maintained the tooth initiation capability of the E10.5 first branchial arches. Representative images of different treatment groups were shown. The dashed box indicated the formation of tooth‐like structures within the transplants. CHIR: CHIR99021, Wnt activator; SAG: Smoothened agonist. DMSO was used as the solvent control. Scale bars: 200 µm. C) Quantitative analysis of the effects of different factors on tooth formation. Only tooth‐like structures with both crown and root would be quantified. Error bars represented data as mean ± SEM from at least four independent experiments: DMSO, *n* = 22; Activin A+SAG, *n* = 17; CHIR+SAG, *n* = 16; Activin A+CHIR, *n* = 21; Activin A+CHIR+SAG, *n* = 16 in 5 days group. DMSO, *n* = 16; Activin A+SAG, *n* = 21; CHIR+SAG, *n* = 14; Activin A+CHIR, *n* = 16; Activin A+CHIR+SAG, *n* = 15 in (5+5) days group. Statistics: *p*‐values were calculated using one‐way ANOVA with Dunnett's test or two‐tailed unpaired Student's *t*‐test by SPSS v27. *p < 0.05, **p < 0.01, ****p < 0.0001. ns: not significant. D) Expressions of markers in odontogenic differentiation confirmed the tooth initiation when co‐stimulated with Activin A and SAG. The samples treated with Activin A and SAG in the (5 + 5) days group were collected on day 0, 3, 5, and 10 for RT‐qPCR analysis. Error bars represented data as mean ± SEM from four independent experiments with triplicates. Statistics: *p*‐values were calculated using two‐tailed unpaired Student's *t*‐test by SPSS v27. ****p < 0.0001.

To do this, the isolated first branchial arches were cultured in the defined medium with or without stimulation for 5 days and part of the samples were then further cultured without any treatment for an additional 5 days before transplantation (**Figure** **4A**). When no stimulants were added, the first branchial arches still grew robustly in the medium but would quickly lose their capability to induce tooth formation (Figure 4B; Figure S6A–C, Supporting Information). Quantitative measurement indicated that by day 10, without stimulants, all the branchial arches would fail to induce tooth formation (Figure 4C, right). However, when Activin A and SAG were added to the medium, most of the primary tissues contained properly developed tooth‐like structures, as revealed by H&E staining (Figure 4B). RT‐qPCR analysis also confirmed the sequential expression of stage‐specific dental markers (Figure 4D), further supporting that the tooth induction within the cultured first branchial arches followed the appropriate developmental path. In addition, we also tested if continuous stimulation with Activin A and SAG would still support the tooth induction. Indeed, tooth‐like structures could also be robustly induced in such culture conditions (Figure S6D–F, Supporting Information). Together, these data indicated that in the defined medium, tooth induction from cultured first branchial arches required additional external factors, and co‐stimulation with Activin A and SAG would help preserve the developmental potential in these tissues during in vitro toothoid culture.

### 2.5 Enamel Induction Required Activation of BMP Signaling

As mentioned above, although differentiated dental lineage cells, such as odontoblasts and ameloblasts, could be readily detected from the cultured toothoids, the deposition of enamel and mature dentin were not observed except a thin layer of pre‐dentin, even when the toothoids were cultured for an extended time (Figure 2G), suggesting that the cells might require additional external signals to start secretion and deposition of the extracellular matrix (ECM) materials over the tooth crown. From research on hair follicle regeneration and full skin reconstruction from human skin stem cells, the air‐liquid interface seemed to play a critical role in inducing appropriate organ polarity and maturation,^[^ ^4^, ^39^ ^]^ which prompted us to speculate that exposure of toothoids to the air‐liquid interface might also promote the ECM deposition and mineralization by ameloblasts and odontoblasts.

**Figure 5:**
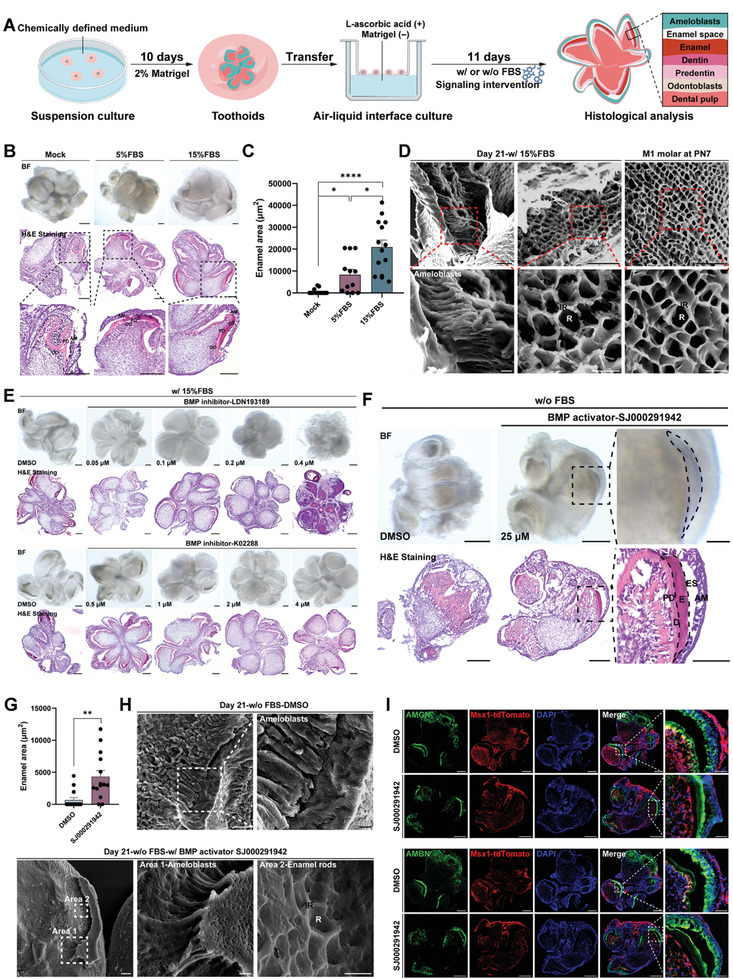
BMP signaling plays an essential role in enamel induction. A) The overall experimental design. The E14.5 toothoids were cultured for 10 days, followed by air‐liquid interface culture for 11 days before histological analysis. B) FBS promoted enamel induction in the toothoids. Serum‐treated toothoids were compared with mock control. Representative images of toothoids stimulated with different serum concentrations were shown (top row). H&E staining confirmed the enamel formation (middle and bottom row). AM: ameloblasts; E: enamel; D: dentin; PD: pre‐dentin; OD: odontoblasts. Scale bars: 200 µm. C) Quantitative analysis of enamel formation (enamel area) in the toothoids. Error bars represented data as mean ± SEM from three independent experiments: Mock, *n* = 14; 5% FBS, *n* = 12; 15% FBS, *n* = 14. Statistics: *p*‐values were calculated using one‐way ANOVA with Dunnett's test by SPSS v27. *p < 0.05, ****p < 0.0001. D) Scanning electron microscopy (SEM) images of elongated prismatic ameloblasts of toothoids versus M1 molar isolated at PN7 stage. The toothoids were analyzed on day 21. The conical indentations were depressions into which Tomes' processes fit to form the enamel rods (R), while the interrod (IR) structure formed through the collaborative secretion of neighboring ameloblasts. Scale bars: 20 µm (top) and 5 µm (zoom‐in). E) Inhibition of BMP signaling strongly suppressed enamel formation in the toothoids. Two BMP inhibitors (LDN193189 and K02288) were used. DMSO served as the solvent control. Scale bars: 200 µm. F) Activating BMP signaling promoted enamel induction. The toothoids were cultured in serum‐free conditions and the DMSO control group would not form enamel. Black dashed lines showed the regions of enamel (zoom in). SJ000291942: a canonical BMP activator; AM: ameloblasts; ES: enamel space; E: enamel; D: dentin; PD: pre‐dentin. Scale bars: 200 µm and 50 µm (zoom‐in). G) Quantitative analysis of the effects of BMP activation on enamel formation. Error bars represented data as mean ± SEM from three independent experiments: *n* = 14 in each group. Statistics: *p*‐value was calculated using two‐tailed unpaired Student's *t*‐test by SPSS v27. **p < 0.01. H) SEM images confirmed the presence of both ameloblasts and enamel in BMP activator‐treated toothoids only, but not in those without the treatment. DMSO served as the solvent control. Scale bars: 20 and 5 µm (zoom‐in). I) BMP activation did not increase the number of ameloblasts and the secretion of enamel matrix proteins. DAPI was used for nuclear staining. AMGN: amelogenin; AMBN: ameloblastin. Scale bars: 200 and 50 µm (zoom‐in).

To do this, toothoids were first cultured in the defined medium before being lifted to the air‐liquid interface for further induction and then histological examination (**Figure** **5**A). Surprisingly, exposure to the air‐liquid interface alone did not stimulate enamel deposition, while serum addition showed dose‐dependent enamel induction (Figure 5B,C). Scanning electron microscopic (SEM) analysis also confirmed that the enamel tissue with normal structure was indeed present (Figure 5D). Further investigation revealed that the serum dependence was not due to the presence of bovine serum albumin (BSA) as BSA could not induce enamel formation either (Figure S7A, Supporting Information), suggesting that other serum factors, most likely growth factors, might play an important role. We then investigated which pathway might be required for enamel induction by using tool compounds to modulate each signaling pathway. Interestingly, neither the blocking of TGF‐β nor Wnt signaling pathways had any significant impact on enamel formation (Figure S7B,C, Supporting Information). However, when BMP signaling was inhibited, either by LDN193189 or K02288 (two BMP inhibitors), enamel induction within the toothoids was strongly inhibited and the effects were dose‐dependent (Figure 5E; Figure S7D, Supporting Information). We then sought to test if activating BMP signaling alone would be able to support enamel formation. Indeed, the addition of SJ000291942, a potent activator of the canonical BMP signaling pathway,^[^ ^40^ ^]^ strongly promoted enamel formation (Figure 5F,G). SEM analysis further confirmed the normal morphology and mineralization of the induced enamel (Figure 5H). E12.5 primary tooth germs and toothoids from other multiple stages (E13.5, E16.5, and E17.5) could also form enamel upon BMP activation (Figure S7E, Supporting Information). Notably, immunostaining of AMGN and AMBN indicated that the expressions of amelogenin and ameloblastin, the major enamel matrix proteins, were similar between BMP activator‐treated samples and non‐treated control (Figure 5I), suggesting that BMP activation did not affect the number of ameloblasts or expression of these enamel matrix proteins, but did stimulate the process of mineralization, thereby promoting enamel deposition. Interestingly, although inhibiting TGF‐β with SB431542 under FBS‐containing conditions did not suppress enamel formation (Figure S7B, Supporting Information), enamel formation was enhanced when toothoids were treated with the TGF‐β1 activator SRI011381^[^ ^41^ ^]^ under chemically defined conditions. Furthermore, synergetic effects were observed when toothoids were treated with both SJ000291942 and SRI011381 (Figure S7F,G, Supporting Information).

Together, these data indicated that enamel formation from the toothoids required stimulation of BMP signaling and the chemically defined culture system could also be used for dissecting the key regulatory mechanisms for tooth ECM deposition and mineralization.

## 3 Discussion

In this study, we developed a chemically defined serum‐free toothoid culture system, which supported tooth reconstitution and continuous development from multiple embryonic stages, and robust differentiation of various dental lineage cells including odontoblasts, ameloblasts as well as periodontal cells. The toothoids also showed prompt response to chemical interventions of key developmental signaling pathways. By using such a system to culture reconstituted tooth germs at different developmental stages (E14.5, E16.5, and E17.5) and systematically characterize stage‐specific dental markers, we discovered that tooth reconstitution did not reset the developmental clock but instead restarted from the original stage when the cells were initially isolated. Second, co‐stimulation of Activin A and SAG would maintain the initial induction of tooth fate from the first branchial arch, which would be otherwise quickly lost in the chemically defined culture. Finally, we showed evidence that activation of BMP/TGF‐β signaling was the critical trigger for ameloblasts to secrete ECM and form mature enamel tissues. Together, these data highlighted the toothoid culture as an important tool for dissecting the molecular mechanisms governing tooth development and regeneration.

Since the first discovery of dental epithelium and mesenchyme would recombine to form a reconstituted tooth^[^ ^7^ ^]^ and a bioengineered tooth germ would restore a lost one in situ,^[^ ^8a,b^ ^]^ the robust reconstitution capabilities of tooth germ cells have prompted the enthusiasm that we may one day grow teeth entirely in a dish. Developmental biology and human pathogenesis studies have provided a breadth of knowledge on the molecular mechanisms governing tooth organogenesis in vivo.^[^ ^2b^ ^]^ However, this knowledge was hardly translated into understanding the tooth reconstitution process, due to the reliance on the traditional approaches of animal models and in vivo transplantation. Compared with hair follicles, which is also a model organ generated through inductive epithelial‐mesenchymal interactions^[^ ^42^ ^]^ and has been induced de novo entirely from human pluripotent stem cells (hPSCs),^[^ ^43^ ^]^ induction and construction of a tooth germ in vitro still faces two challenges: i) a thorough dissection of key cell populations and their roles in tooth development to establish a practical route for induced differentiation from hPSCs; ii) a defined culture system that could support tooth development and maturation. For the first challenge, with the advancement and application of single‐cell sequencing and spatial profiling, the functional roles of different cell compositions during tooth development and homeostasis started to be revealed.^[^ ^14^, ^15^ ^]^ Among these studies, it was recently reported that human pre‐ameloblasts could be directly induced from pluripotent stem cells but lack of evidence for the induction of natural enamel.^[^ ^14^, ^16^ ^]^ In addition, for organoid culture, recent progress indicated that primary dental epithelial cells could be readily maintained in 3D cultures and retained the capability to further differentiate into ameloblast‐like cells,^[^ ^16d^ ^]^ however, the epithelial organoid alone could only partially express matrix proteins such as AMGN without clear mineralized enamel formation/deposition. Adult dental epithelial cells were also explored using organoid culture and shown to deposit hydroxylapatite crystals upon in vivo transplantation under kidney capsules.^[^ ^16b^ ^]^ Meanwhile, it is still very challenging to maintain the self‐renewal of primary dental mesenchymal progenitors alone and the tooth‐inductive potential would be quickly lost in such a culture system,^[^ ^44^ ^]^ suggesting epithelial signals may be critical in maintaining such potential and continuous tooth development. Therefore, the work presented here, on one hand, would be a step forward to support full‐spectrum tooth organoid development and serve as a valuable tool for dissecting the principles behind tooth reconstitution and the roles of various cell compositions in driving such process. On the other hand, it also offers an essential culture microenvironment for tooth bioengineering, which may support further exploration of inducing both dental epithelial and mesenchymal lineages directly from pluripotent stem cells.

The discovery of stimulating BMP/TGF‐β signaling would trigger the deposition of enamel by ameloblasts revealed an interesting role of BMP/TGF‐β signaling in mineralized tissue biology. Previously, animal studies using conditional knockout mice have demonstrated that BMP, TGF‐β, and Wnt signaling were all required for proper lineage differentiation of ameloblasts and enamel formation in vivo.^[^ ^45^ ^]^ Our data did not contradict these results but instead provided a more in‐depth view of how the enamel mineralization was regulated. In the chemically‐defined culture, the lineage specification of ameloblasts did not require any additional BMP, TGF‐β or Wnt stimulation from the microenvironment (medium), indicating that the intrinsic signaling activities supplied through the inductive interactions between dental epithelium and mesenchyme were already sufficient to drive the differentiation process. However, triggering the enamel deposition required an extra boost of BMP/TGF‐β signaling. Interestingly, this is consistent with the natural course of postnatal enamel development (PN3).^[^ ^46^ ^]^ Mechanical loading (milk sucking perhaps) at such a stage is likely to be the key factor as it is evident to activate BMP/TGF‐β signaling during bone development and remodeling.^[^ ^47^ ^]^

Finally, it is worth noting that the current toothoid culture is still far from perfection. For example, it still relied on primary cells and would not be able to keep the toothoid in a proliferating state but instead support the continuous differentiation of dental lineages. In addition, it could not induce root development in vitro either, which is an important process for postnatal tooth establishment. However, previous explorations of tooth reconstitution using serum‐containing medium^[^ ^8^, ^48^ ^]^ and our study all supported that the reconstituted toothoid retained the full potential for root development, as it could readily develop once being transplanted in vivo under kidney capsule or alveolar bone, suggesting that the additional signals from microenvironment might be essential. Interestingly, recent work in stomach organoids indicated that stomach epithelium could coordinate with neural crest cells to form organoids with surrounding smooth muscle layers.^[^ ^49^ ^]^ Like smooth muscle, alveolar bones are also formed by neural crest‐derived cells, and thus further improvement by combining the toothoid with neural crest cells may help to generate a fully developed tooth in vitro.

## 4 Experimental Section

### Ethical Approval

All animal experiments were approved by the Institutional Animal Care and Use Committee at the College of Life Sciences, Sichuan University (Approval#: SCU2203009 and 20200401001).

### Mouse Strains and Animal Care

Animals were maintained under standardized conditions including a regulated 12‐h light/dark cycle and stable temperature. They were kept in individually ventilated cages with at least one companion mouse and had unrestricted access to both food and water. After weaning, mice older than 8 weeks were selected for mating, with the appearance of vaginal plugs used to designate embryonic day E0.5. To more accurately determine the developmental stage of embryos, additional morphological assessments were conducted, including comparing the sizes of the maxillary and mandibular primordia. Embryo samples were collected at specific time points as described in the main text. Knock‐in transgenic mice (Pitx2^P2A‐copGFP^ and Msx1^P2A‐tdTomato^) were generated by Biocytogen, Inc. (Beijing).

### Tooth Germ Isolation and Dissociation

The molar and incisor tooth germs were physically separated from the mandible using dissection needles under a stereo‐fluorescent microscope (Olympus, SZX10, Japan). The surrounding tissues of the tooth germs were removed as thoroughly as possible, and the number of tooth germs was counted before being collected into a 1.5 mL microcentrifuge tube. Next, the tooth germs were washed twice with PBS (Gibco, Cat# C10010500BT) and dissociated with 0.5 mL 0.25% Trypsin‐EDTA (ThermoFisher, Cat# 25200072) for 5–15 min (E12.5, 5 min; E13.5, 5 min; E14.5, 7 min; E16.5, 10 min; E18.5, 12 min; PN1, 15 min) in an incubator at 37 °C with 5% CO_2_. After that, 0.5 mL serum‐containing medium (DMEM (Gibco, Cat# C11995500BT), 10% v/v fetal bovine serum (FBS) (Hyclone, Cat# SH30084.03), Penicillin/Streptomycin (Pen‐Strep) (Gibco, Cat# 15140‐122) supplied with 20 µL 5 mg·mL^−1^ DNase I (Roche, Cat# 10104159001) was used to neutralize the enzymes and prevent cell aggregates. Finally, single‐cell suspensions were further filtered through a 70 µm strainer (Corning, Cat# 352350).

### Tooth Germ Reconstitution In Vitro

The single‐cell suspension was divided into 1.5 mL microcentrifuge tubes according to the number of isolated tooth germs, ensuring that the exact number of cells and the ratio of epithelial to mesenchymal cells remained consistent with those in the primary tooth germs (Table S2, Supporting Information). Next, two rounds of centrifugation at 3000 g for 4 min were performed. The first round collected the cell pellet, while the second was performed to coat the pellet with 15 µL 2 mg·mL^−1^ collagen gel. The microcentrifuge tubes were then placed in an incubator at 37 °C with 5% CO₂ for 30 min to allow the collagen gel to solidify. Afterward, the collagen gel‐coated cell pellet was transferred with culture medium into the 24‐well culture plates (BIOFIL, Cat# TCP011024) pre‐treated by 2% w/v PolyHEMA (2‐hydroxyethyl methacrylate) (Sigma–Aldrich, Cat# P3932) at the density of 2–3 cell pellets per well with 1 mL culture medium.

### Toothoid Culture

The reconstituted toothoids (E13.5‐PN1) and natural E12.5 tooth germs without reconstitution were cultured in the chemically defined medium (Glasgow's MEM (GMEM) (Gibco, Cat# 11710035), 1.5% v/v Knock‐Out Serum Replacement (KSR) (Gibco, Cat# 10828028), NEAA (Gibco, Cat# 11140050), Pen‐Strep (Gibco, Cat# 15140122), Sodium pyruvate (Gibco, Cat# 11360070), 0.1 mM 2‐Mercaptoethanol (Sigma‐Aldrich, Cat# M3148) containing 2% v/v Matrigel (Corning, Cat# 354230) (Table S1B, Supporting Information). Half of the medium (500 µL) was refreshed every other day. The morphology and fluorescence of the toothoids could be observed directly under the fluorescence microscope (Olympus, IX73, Japan).

### E10.5 First Branchial Arch Isolation and Organ Culture

The rostral regions of E10.5 first branchial arches were physically separated from the mandible using dissection needles under a stereo‐fluorescent microscope. Every 2–3 tissues were cultured in 1 mL chemically defined medium containing various combinations of stimulus molecules, including Activin A (10 ng·mL^−1^, Solarbio, Cat# P00101), CHIR99021 (3 µm, MCE, Cat# HY‐10182) and SAG (200 nm, MCE, Cat# HY‐12848) into the 24‐well culture plates pre‐treated by 2% w/v PolyHEMA. The first branchial arches were kept in the defined medium containing small molecules for 5 days (the “5 days” group) or switched to the defined medium without compounds from day 5 and cultured in vitro for an additional 5 days (the “5 + 5 days” group) before kidney capsule transplantation. In the “10 days” group, the first branchial arches were cultured in the chemically defined medium with continuous compound treatment for 10 days before transplantation. Half of the medium (500 µL) was refreshed every other day.

### Enamel Induction In Vitro

The E14.5 toothoids were suspension cultured in chemically defined medium containing 2% v/v Matrigel for 10 days before carefully transferred on a 70 µm strainer (Corning, Cat# 352350) in a six‐well culture plate (BIOFIL, Cat# TCP011006), and then added 4 mL defined medium (without Matrigel) containing 100 µg·mL^−1^ L‐ascorbic acid (Sigma–Aldrich, Cat# A4403) for air‐liquid interface culture. To explore the induction of enamel deposition, different concentrations of FBS (5% and 15% v/v) were used, or various small molecules, including 0.05–0.4 µm LDN193189 (MCE, Cat# HY‐12071A), 0.5–4 µm K02288 (MCE, Cat# HY‐12278), 5–10 µm SB431542 (MCE, Cat# HY‐10431) or 0.5–1 µm XAV939 (MCE, Cat# HY‐15147) were supplemented under 15% FBS conditions. SJ000291942 (25 µm, MCE, Cat# HY‐112331) and SRI011381 (10 µm, MCE, Cat# HY‐100347) were supplemented without FBS. Half of the medium (2 mL) was refreshed every 3 days. After 21 days, enamel formation was identified by H&E staining following the manufacturer's protocol (SOLARBIO, Cat# G1120).

### Statistical Analysis

All the statistical analysis was performed with IBM Statistical Package for the Social Sciences (SPSS) version 27.0 (IBM Corp, Armonk, NY, USA). Detailed statistical analysis was specified in each figure legend. All data were expressed as mean ± standard error of the mean (SEM). The data of different groups were compared using unpaired two‐tailed Student's *t*‐test or one‐way ANOVA followed by Dunnett's post‐hoc test. *p* < 0.05 was deemed statistically significant, and different *p*‐values were indicated by *(*p* < 0.05), **(*p* < 0.01), ***(*p* < 0.001), ****(*p* < 0.0001), and ns indicated not significant.

## Conflict of Interest

The authors declare no conflict of interest.

## Author Contributions

Z.L. and Z.Z. conceived the study. Z.Z., H.H., and Z.X. performed most of the wet experiments. H.C. performed the bioinformatic analysis. C.S., K.X., K.W., Y.W., and Q.Z. provided experimental assistance. Z.L., H.C., Y.Z., and Y.Y. oversaw the collection of results and data interpretation. Z.L. and Z.Z. wrote the manuscript. All authors have seen and approved the final version of the paper.

## Supporting information



Supporting Information

## Data Availability

The data that support the findings of this study are available in the supplementary material of this article.
