# Supplementary material for: A Chemically Defined Culture for Tooth Reconstitution
Source: Adv Sci (Weinh). 2024 Nov 27;12(3):2404345. doi: 10.1002/advs.202404345 (PMC11744639; doi:10.1002/advs.202404345)
Supplement: Supplementary file 1 — Supporting Information [file ADVS-12-2404345-s001.docx]

**Supplemental methods**

**Collagen gel preparation:** The collagen gel used for tooth germ reconstitution was primarily prepared according to the manufacturer’s protocol for PureCol Type I collagen solution (Advanced Biomatrix, Cat# 5005), with necessary optimizations for toothoid formation. Briefly, one part of chilled 10× MEM (Gibco, Cat# 61100061) was slowly added to eight parts of chilled collagen solution with gentle swirling, and the pH of the mixture was adjusted to 6.5-7.0 using sterile 1 M NaOH (Sigma-Aldrich, Cat# 655104). Three parts of chilled DMEM (Gibco, Cat# C11995500BT) were then added to the mixture with gentle swirling, resulting in a final collagen concentration of 2 mg·mL^-1^. To prevent gelation, the temperature of the mixture was maintained at 2-10 ℃.

**Small molecule compounds preparation:** All inhibitors, activators, and agonists were purchased in powder form from MedChemExpress (MCE) and diluted in Dimethyl sulfoxide (DMSO) (Sigma-Aldrich, Cat# D4540) according to the manufacturer’s protocol. The effectiveness of the small molecule compounds at key concentrations was validated by measuring the relative expression levels of downstream target genes using RT-qPCR (**Figure S8**).

**Renal capsule transplantation:** The toothoids were isolated from collagen gel and transplanted into the left renal capsule of 8-week-old male C57BL/6JGpt mice (# N000013, GemPharmatech Co., Ltd, Chengdu, China). Up to 4 samples were transplanted per mouse. The grafts were retrieved 2-4 weeks post-transplantation, fixed in 4% w/v paraformaldehyde (PFA) (Sigma-Aldrich, Cat# 158127-500G), and subjected to Micro-CT scanning. Alternatively, they were decalcified in 10% w/v EDTA (SOLARBIO, Cat# E8040) for 2 days before further analysis.

**Frozen section and histological evaluations of tissues:** All the 2-week grafts and mouse embryos were fixed in 4% PFA at 4 ℃ overnight and decalcified in 10% w/v EDTA at room temperature (RT) for 2 days before dehydration by 15% and 30% w/v sucrose solution until sunk and then pre-treated in the mixture of 30% sucrose solution and OCT (optimal cutting temperature, SAKURA, Cat# 4583) for 1 hour before final embedding in the OCT and placed at -80 ℃ for 24 hours. The toothoids cultured *in vitro* were fixed in 4% PFA at 4 ℃ for 30 min and dehydrated in 15% and 30% sucrose solution until sunk before embedding in the OCT and placed at -80 ℃ for 2 hours. All the samples were sectioned at 6 μm thickness with the microtome cryostat (Leica CM1950). Tissue sections were stored at -20 ℃ prior to histological analysis. For the histological staining, the sections were rehydrated in PBS for 10 min to eliminate the OCT compound, followed by H&E staining according to the manufacturer's protocol. Brightfield images were captured using a digital panoramic scanner (Wisleap WS-10).

**Immunofluorescence and RNAscope for tissue sections:** For immunofluorescence staining, frozen sections were rehydrated in PBS for 10 min to remove the OCT compound. For intracellular antigens, sections were permeabilized with 0.3% Triton X-100 (Sigma-Aldrich, Cat# T9284-100ML) for 5 min and washed in PBS for 5 min. Subsequently, the sections were treated with a blocking solution (5% w/v BSA (Sigma-Aldrich, Cat# A3311-10G) in PBS) at RT for 1 hour and incubated overnight at 4 °C with primary antibodies diluted in the blocking solution. Secondary antibodies were incubated at RT for 1 hour, protected from light, and then washed three times with PBS before being mounted with 4’,6-diamidino-2-phenylindole (DAPI) (ThermoFisher, Cat# D1306) mounting medium. All sections were examined using either an Olympus VS200 slide scanner or a confocal laser scanning microscope (Leica TCS SP5II). RNAscope staining process was conducted according to the manufacturer’s instructions (ACDbio, Cat# 323100). Following the RNAscope staining, immunoﬂuorescence staining was performed as described above. All the antibodies and RNAscope probes were listed in **Table S3**.

**Micro-computed tomography (Micro-CT) analysis**: The 4-week grafts were radiographed using μCT50 system (Scanco Medical, Bassersdorf, Switzerland) with a spatial resolution of 5 μm (70 kVp, 200 μA, 300 ms integration time). 3D imaging reconstruction was performed after defining the region of interest within a threshold range of 220-1000 using the accompanying Micro-CT system software.

**Scanning electron microscopy (SEM) analysis:** The toothoids induced for enamel deposition were washed twice in PBS and the epithelial tissues surrounding the enamel were eliminated to expose the enamel surface before being fixed in 2.5% glutaraldehyde solution (Kermel, Cat# 111-30-8) at 4 °C for 2 hours, followed by a double rinse in PBS, and then gently dehydrated in sequential ethanol gradient (15 min in each of 30, 50, 60, 70, 80, 90, and 100%). Subsequently, the toothoids were air-dried in a bottom heating oven (Labwit, ZXFD-B5090). After sputtering with gold and palladium, the samples were examined using the SEM (Thermo Fisher Scientific, Apreo S HiVoc) operated at 15 kV.

**Quantitative PCR:** Total RNAs were extracted from cells using TRI Reagent (Sigma-Aldrich, Cat# T9424) and reverse-transcribed to cDNA with the PrimeScript RT reagent kit with gDNA Eraser (TAKARA, Cat# RR047A) following the manufacturer's protocol. Quantitative PCR was performed on a CFX384 Optics Module (Bio-Rad, USA) using AceQ Universal SYBR qPCR Master Mix (Vazyme, Cat# Q511-02), and relative expression levels were calculated using the comparative threshold cycle (∆∆CT) method. All experiments were performed in triplicate. Primer sequences are provided in **Table S4**.

**scRNA-seq data analysis:** The scRNA-seq data for the first branchial arches at E10.5 and molars at E12.5, E14.5, E16.5, and PN1 were obtained and analyzed as described previously^1^. The heatmap of stage-specific genes was performed using R and Seurat v4.0 based on the average gene expression levels.

**References:**

1 Hu, H. *et al.* Dental niche cells directly contribute to tooth reconstitution and morphogenesis. *Cell Rep* **41**, 111737, doi:10.1016/j.celrep.2022.111737 (2022).

**A chemically defined culture for tooth reconstitution**

**Supplemental Figure Legend**

**Figure S1. Monitoring self-organization and organ formation of the reconstituted** **toothoids using the dual color mouse model.**

**(A)** Representative images showing specific labeling of molar dental epithelium and mesenchyme at different developmental stages by copGFP (Pitx2) and tdTomato (Msx1) respectively. DAPI was used for nuclear staining. Scale bars: 100 μm. **(B)** Representative time course images of reconstituted toothoids cultured *in vitro*. The self-reorganization, growth, and differentiation processes could be closely monitored through fluorescence-labeled cells. Scale bars: 200 μm. **(C)** The reconstituted toothoids were able to develop into tooth-like structures upon transplantation. Multiple tooth-like structures could be observed after two weeks of transplantation. AM: ameloblasts; E: enamel; D: dentin; PD: pre-dentin; OD: odontoblasts; P: dental pulp; BV: blood vessel; PDL: periodontal ligament tissues. Scale bars: 500 μm (left) and 200 μm (middle and right). **(D)** MicroCT analysis confirmed the formation of calcified structures in the samples transplanted for four weeks. Tooth morphology was presented with the 3D reconstructed image. Scale bars: 500 μm (left) and 200 μm (right).

**Figure S2. The defined culture system supported the development of toothoids reconstituted from multiple stages.**

**(A)** Representative images of tooth germs isolated from multiple developmental stages (E12.5-PN1). Tooth germs were isolated from the Msx1^P2A-tdTomato^ mice. Scale bars: 500 μm. **(B)** Time course images of toothoids cultured in the chemically defined medium. The progressions of self-reorganization, growth, and differentiation were closely monitored. Scale bars: 200 μm. **(C)** The toothoids still retained the capability to develop into tooth-like structures upon transplantation *in vivo*. Reconstituted tooth germs from E13.5 to E18.5 could form tooth-like structures upon transplantation, while those from E12.5 or PN1 could not. Scale bars: 500 μm (left) and 200 μm (middle and right). **(D)** The E12.5 tooth germs directly cultured *in vitro* for 10 days would maintain tooth-forming capability upon transplantation *in vivo*. H&E staining of frozen sections from the 2-week samples confirmed the formation of tooth-like structures. AM: ameloblasts; ES: enamel space; E: enamel; D: dentin; PD: pre-dentin; OD: odontoblasts; P: dental pulp; BV: blood vessel. Scale bars: 200 μm.

**Figure S3. The defined culture system also supported the development of incisor toothoids.**

**(A)** Representative images showing specific labeling of incisor dental epithelium and mesenchyme at E14.5 by copGFP (Pitx2) and tdTomato (Msx1) respectively. Scale bars: 500 μm. **(B)** Representative time course images of reconstituted E14.5 incisor toothoids cultured *in vitro*. The self-reorganization, growth, and differentiation processes of incisor toothoids could be closely monitored through fluorescence-labeled cells. Scale bars: 200 μm. **(C)** The incisor toothoids maintained the capability to develop into tooth-like structures upon transplantation *in vivo*. Multiple tooth-like structures were observed 2 weeks post-transplantation. AM: ameloblasts; E: enamel; D: dentin; PD: pre-dentin; OD: odontoblasts; P: dental pulp; BV: blood vessel. Scale bars: 500 μm (left) and 250 μm (right). **(D)** Quantitative analysis and comparison of the number (left) and size (right) of formed tooth-like structures between molar and incisor toothoids. Only toothoids with both crown and root would be quantified. Error bars represented data as mean ± SEM from three independent experiments: number, n = 24; maximum area, n = 21. Statistic: *p*-values were calculated using two-tailed unpaired Student’s *t*-test by SPSS v27. *****p* < 0.0001. **(E)** Representative images of incisor tooth germs isolated from multiple developmental stages (E13.5-PN1). Tooth germs were isolated from the Msx1^P2A-tdTomato^ mice. Scale bars: 500 μm. **(F)** The incisor toothoids still retained the capability to develop into tooth-like structures upon transplantation *in vivo*. Reconstituted incisor toothoids from E13.5 to E17.5, but not 18.5 and PN1, could form tooth-like structures upon transplantation. AM: ameloblasts; ES: enamel space; E: enamel; D: dentin; PD: pre-dentin; OD: odontoblasts; P: dental pulp. Scale bars: 200 μm. **(G)** The absence of enamel and ameloblasts in incisor toothoids at E16.5 and later stages. The formation of enamel and ameloblasts in incisor toothoids was analyzed by immunostaining for AMGN, while the presence of odontoblasts was confirmed by immunostaining for NESTIN. DAPI was used for nuclear staining. Scale bars: 200 μm.

**Figure S4. Recapitulating tooth development by toothoids derived from the chemically defined culture system.**

**(A)** Immunostaining of SOX9 in toothoids. The E13.5 toothoids were cultured *in vitro* for 10 days. Representative images showed that SOX9 expression was observed in the dental papilla region on day 6. Epi: epithelium; DP: dental papilla. Scale bars: 100 μm and 50 μm (zoom-in). **(B)** Immunostaining and direct comparison of SP7 expression in the toothoids vs M1 molars at different developmental stages. The E14.5 toothoids were cultured *in vitro* for 21 days. The expression of SP7 gradually becomes higher in the odontoblasts (white dashed line) compared to other regions. DAPI: nuclear staining. Scale bars: 100 μm. **(C)** The derivation of dental lineage cells was observed in toothoids from multiple stages. The E12.5 primary tooth germs, as well as the E13.5, E16.5, and E17.5 toothoids, were cultured *in vitro* and analyzed on day 24. The formation of ameloblasts and odontoblasts in the toothoids was confirmed by H&E staining, RNAscope, and immunostaining. AM: ameloblasts; PD: pre-dentin; OD: odontoblasts; P: dental pulp; Enamel proteins: AMGN, AMBN; Odontoblasts: NESTIN; dentin sialophosphoprotein: *Dspp*. Scale bars: 100 μm. **(D)** The reconstituted toothoids treated with small molecules lose the capability to self-organize *in vitro*. The reconstituted toothoids were treated with 10 μM SB431542 (TGF-β inhibitor), 0.5 μM XAV939 (Wnt inhibitor), or 3 μM CHIR99021 (Wnt activator) for 10 days. DMSO was used as the solvent control. Scale bars: 200 μm. **(E)** Dose-dependent inhibition of tooth reconstitution by small molecules. The reconstituted toothoids were treated with 3-20 μM SB431542, 0.1-1 μM XAV939, or 0.5-5 μM CHIR99021 for 10 days before transplantation for two weeks. DMSO was used as the solvent control. H&E staining of frozen sections confirmed the effects of small molecules on tooth formation. Scale bars: 250 μm. **(F)** Immunostaining with anti-K14 antibody indicated the formation of multiple epithelial pearls in SB431542 and XAV939 treated groups. DAPI: nuclear staining. K14, keratin 14. Scale bars: 250 μm.

**Figure S5. Tooth reconstitution did not reset the developmental clock.**

**(A-B)** Immunostaining of stage-specific SOX9 and SDC1 in the E17.5 toothoids. The toothoids were cultured for 7 days. SOX9 expression was observed in the dental papilla region on day 2, while SDC1 was no longer expressed in the dental papilla region after day 4. DAPI was used for nuclear staining. Epi: epithelium; DP: dental papilla. Scale bars: 100 μm. **(C)** Analysis of stage-specific dental markers in E17.5 toothoids by RT-qPCR. Error bars represented data as mean ± SEM from at least two independent experiments with triplicates. **(D)** RT-qPCR Analysis of stage-specific dental markers in E14.5 toothoids indicated that the reconstituted toothoids did not restart from the initiation stage. Markers of early development stages (*Hand1*, *Hand2*, and *Fgf8*) were not expressed throughout the culture process. Error bars represented data as mean ± SEM from at least two independent experiments with triplicates.

**Figure S6. Tooth initiation of the first branchial arches required co-stimulation with Activin A and Smoothhead agonist (SAG).**

**(A)** The first branchial arch at E10.5 would quickly lose tooth induction capability during the *in vitro* culture. The rostral regions of E10.5 first branchial arches were either transplanted immediately or cultured in the chemically defined medium for 10 days before transplantation. H&E staining of frozen sections confirmed the loss of tooth formation. Scale bars: 200 μm. **(B)** Time course images of different treatment conditions for first branchial arch cultured *in vitro*. CHIR: CHIR99021, Wnt activator; SAG: Smoothhead agonist. DMSO was used as the solvent control. Scale bars: 200 μm. **(C)** Representative images of grafts with different treatment conditions were harvested after two weeks. The DMSO-treated sample was used as the control. Scale bars: 500 μm. **(D)** Schematic experimental strategy. The rostral regions of E10.5 first branchial arches were cultured in the chemically defined medium and induced continuously with small molecules and growth factors for 10 days before transplantation. **(E)** Continuous co-stimulation with Activin A and SAG maintained the tooth formation capability of the E10.5 first branchial arches. Representative images of different treatment conditions were shown. DMSO was used as the solvent control. Scale bars: 500 μm (grafts); 200 μm (organ culture and H&E Staining). **(F)** Quantitative analysis of the effects of continuous stimulation with different factors on tooth formation. Only tooth-like structures with both crown and root would be quantified. Error bars represented data as mean ± SEM from four independent experiments: DMSO, n = 15; Activin A+SAG, n = 14; CHIR+SAG, n = 12; Activin A+CHIR, n = 12; Activin A+CHIR+SAG, n = 13. Statistics: *p*-values were calculated using one-way ANOVA with Dunnett’s test by SPSS v27. **p* < 0.05. ns: not significant.

**Figure S7. Activation of BMP and TGF-β signaling exhibited synergetic effects on enamel formation.**

**(A)** BSA alone did not promote enamel induction in toothoids. Scale bars: 200 μm. **(B-C)** Inhibition of TGF-β and Wnt signaling did not affect enamel induction by FBS. SB431542: a TGF-β inhibitor; XAV939: a Wnt inhibitor. DMSO was used as the solvent control. Scale bars: 200 μm. **(D)** Quantitative analysis of the enamel area indicated that BMP inhibitors strongly suppressed enamel formation in the toothoids. LDN193189 and K02288: BMP inhibitors. DMSO served as the solvent control. Error bars represented data as mean ± SEM from two independent experiments with triplicates. Statistics: *p*-values were calculated using two-tailed unpaired Student’s *t*-test or one-way ANOVA with Dunnett’s test by SPSS v27. *****p* < 0.0001. **(E)** Activating BMP signaling stimulated enamel formation in toothoids from multiple stages. The E12.5 primary tooth germs and the E13.5, E16.5, and E17.5 toothoids were tested. Black dashed lines indicated the regions of enamel (zoom-in). SJ000291942: a canonical BMP activator; AM: ameloblasts; ES: enamel space; E: enamel; D: dentin; PD: pre-dentin. DMSO served as the solvent control. Scale bars: 100 μm. **(F)** Co-stimulation of BMP and TGF-β1 signaling showed synergetic effects on enamel formation. The E14.5 toothoids were treated with small molecules. SJ: SJ000291942, a canonical BMP activator; SRI: SRI011381, a TGF-β activator. DMSO served as the solvent control. Scale bars: 200 μm. **(G)** Quantitative analysis of enamel formation (enamel area) in the treated toothoids. Error bars represented data as mean ± SEM from three independent experiments: n = 14 in each group. Statistics: *p*-values were calculated using one-way ANOVA with Dunnett’s test by SPSS v27. ***p* < 0.001.

**Figure S8. Confirmation of the effective treatment by small molecules at the respective concentrations.**

**(A)** SB431542 (TGF-β inhibitor), XAV939 (Wnt inhibitor), and CHIR99021 (Wnt activator) used in E14.5 toothoids culture were effective. The E14.5 toothoids treated with SB4315421 (10 μM), XAV939 (0.5 μM), or CHIR99021 (3 μM) *in vitro* were analyzed at day 10. Error bars represented data as mean ± SEM from at least two independent experiments with triplicates. Statistics: *p*-values were calculated using two-tailed unpaired Student’s *t*-test by SPSS v27. ***p* < 0.01, ****p* < 0.001, *****p* < 0.0001. **(B)** Expression of SHH downstream target genes confirmed that the SAG (smoothened agonist) used in E10.5 first branchial arch organ culture was effective. The E10.5 first branchial arches treated with SAG (0.2 μM) were analyzed on day 5. Error bars represented data as mean ± SEM from at least two independent experiments with triplicates. Statistics: *p*-values were calculated using two-tailed unpaired Student’s *t*-test by SPSS v27. *****p* < 0.0001**. (C)** Expression of TGF-β, Wnt/β-catenin, and BMP downstream target genes confirmed that the SB431542, XAV939, LDN193189 (a BMP inhibitor), K02288 (a BMP inhibitor), SJ000291942 (a BMP activator), and SRI011381 (a TGF-β activator) used in E14.5 toothoids enamel inhibition or induction were effective. The E14.5 toothoids treated with SB431542 (10 μM), XAV939 (0.5 μM), LDN193189 (0.4 μM), K02288 (4 μM), SJ000291942 (25 μM), and SRI011381 (10 μM) were analyzed on day 21. Error bars represented data as mean ± SEM from at least two independent experiments with triplicates. Statistics: *p*-values were calculated using two-tailed unpaired Student’s *t*-test by SPSS v27. **p* < 0.05, ***p* < 0.01, ****p* < 0.001.*****p* < 0.0001.


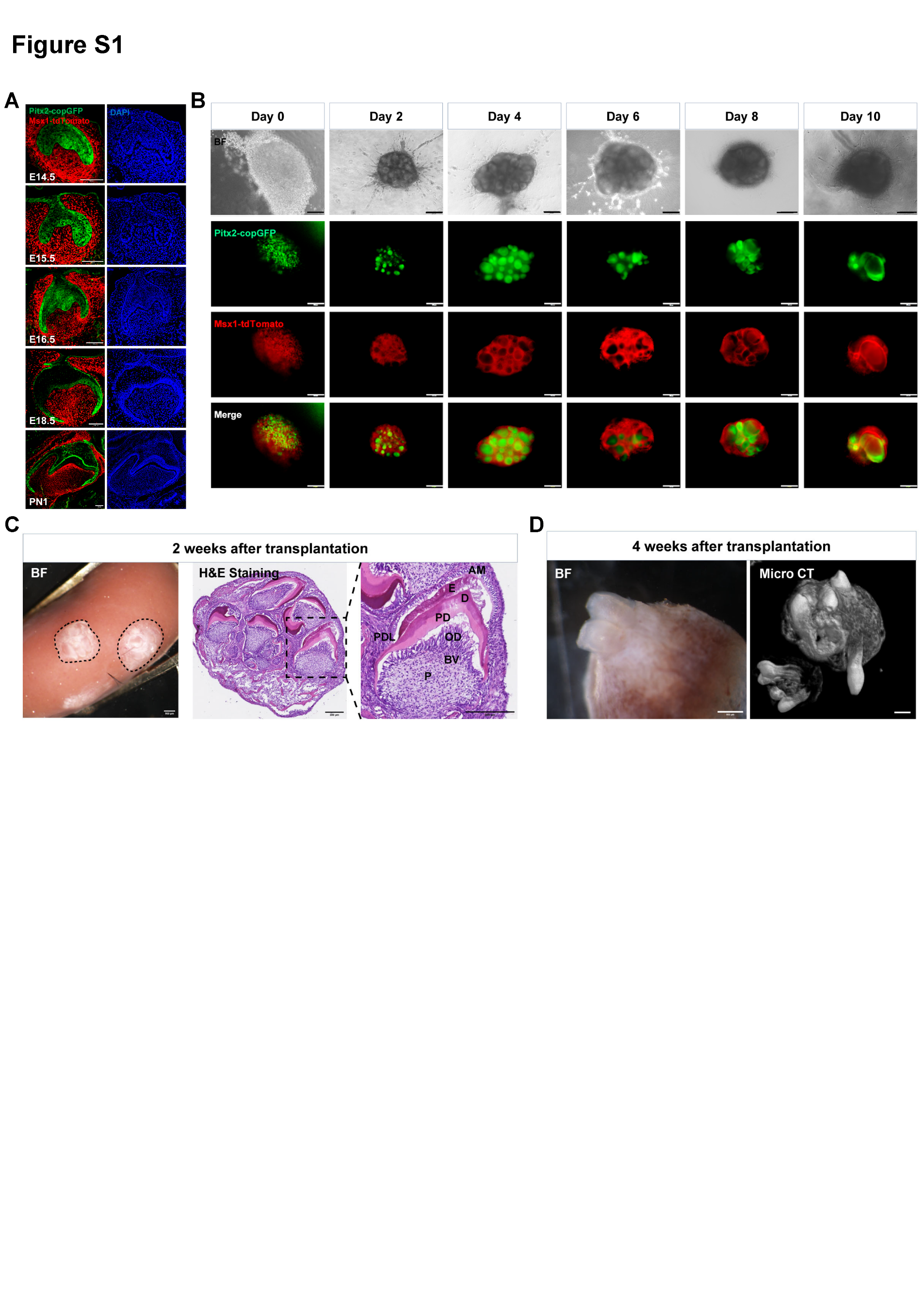

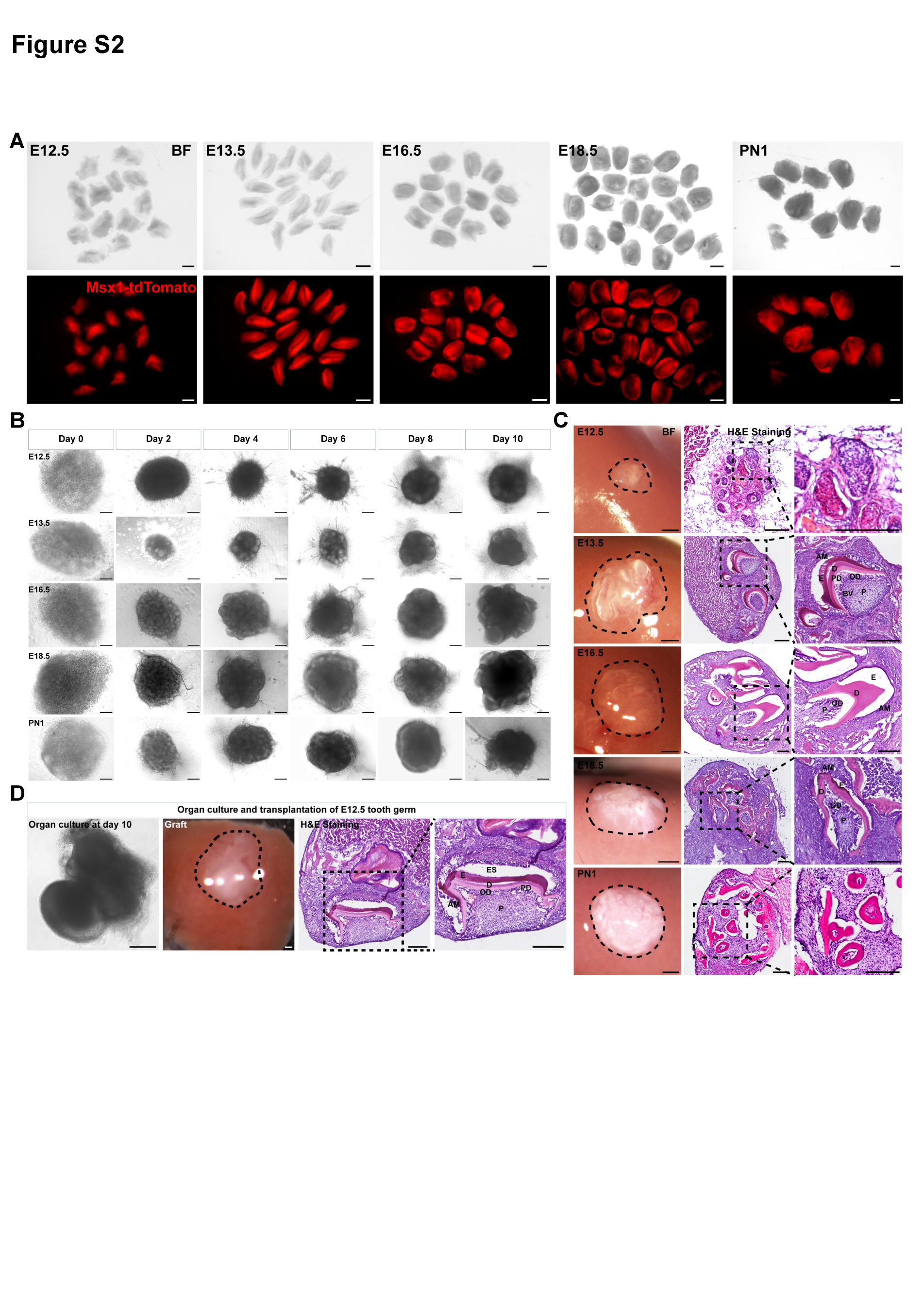

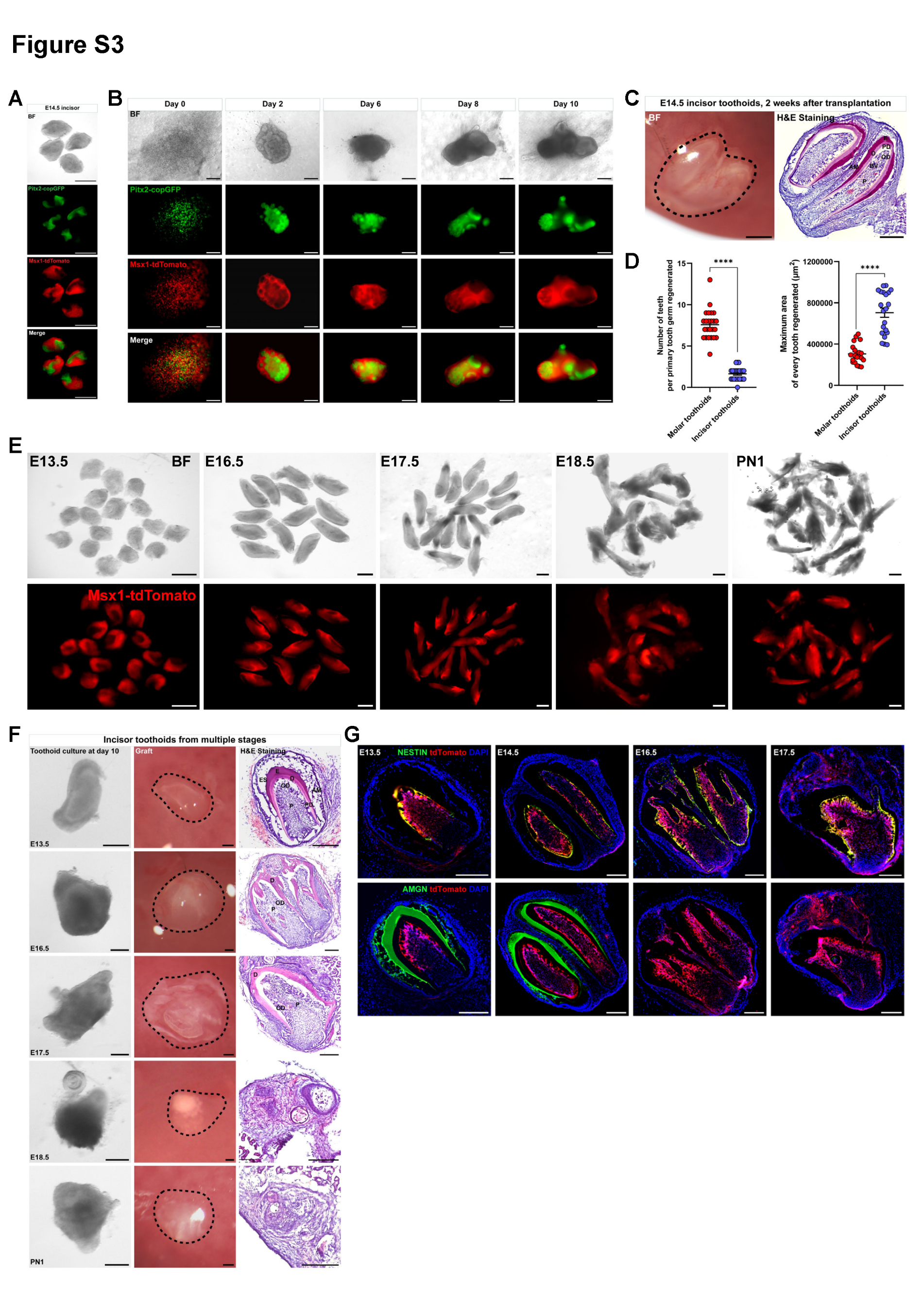

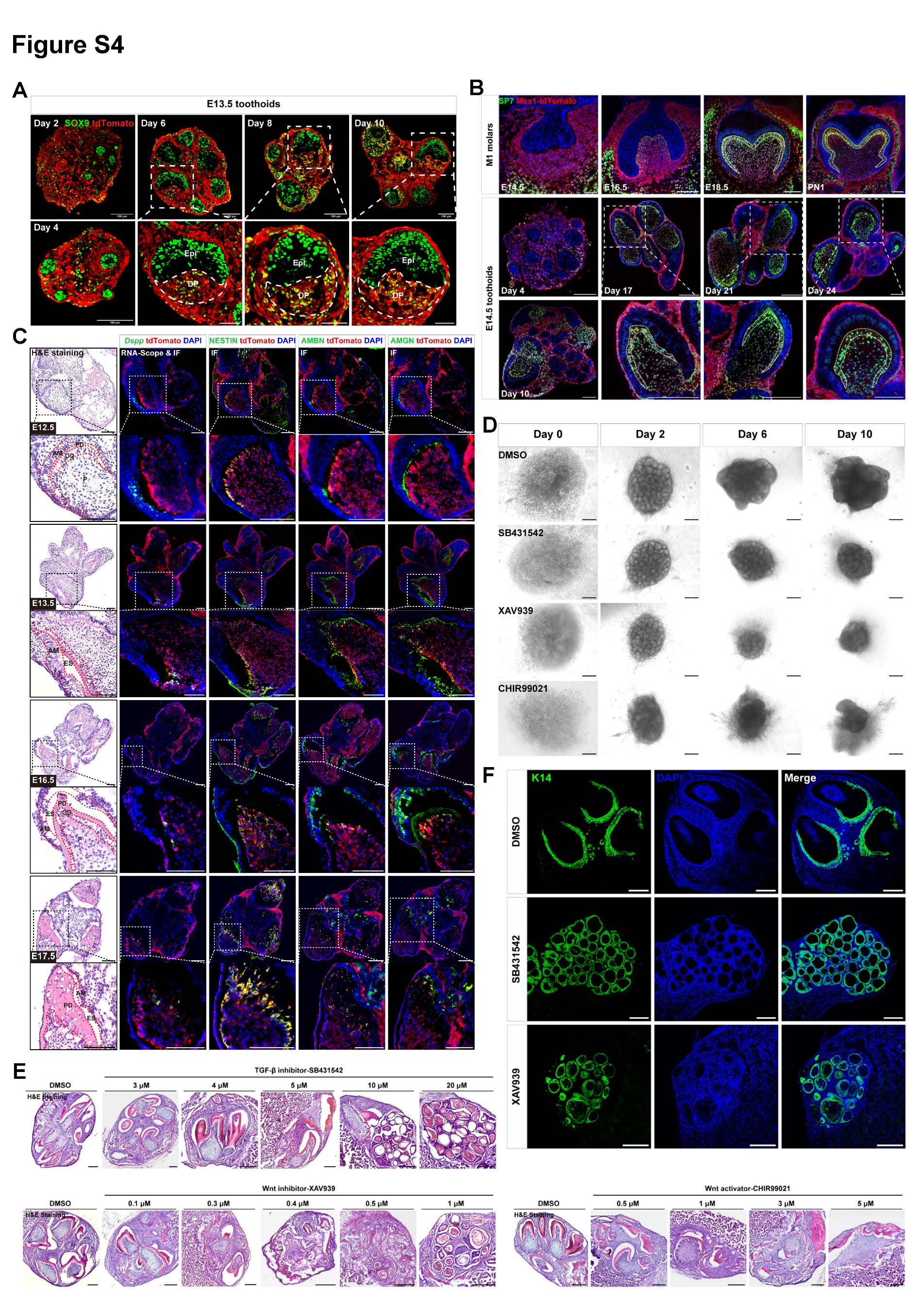

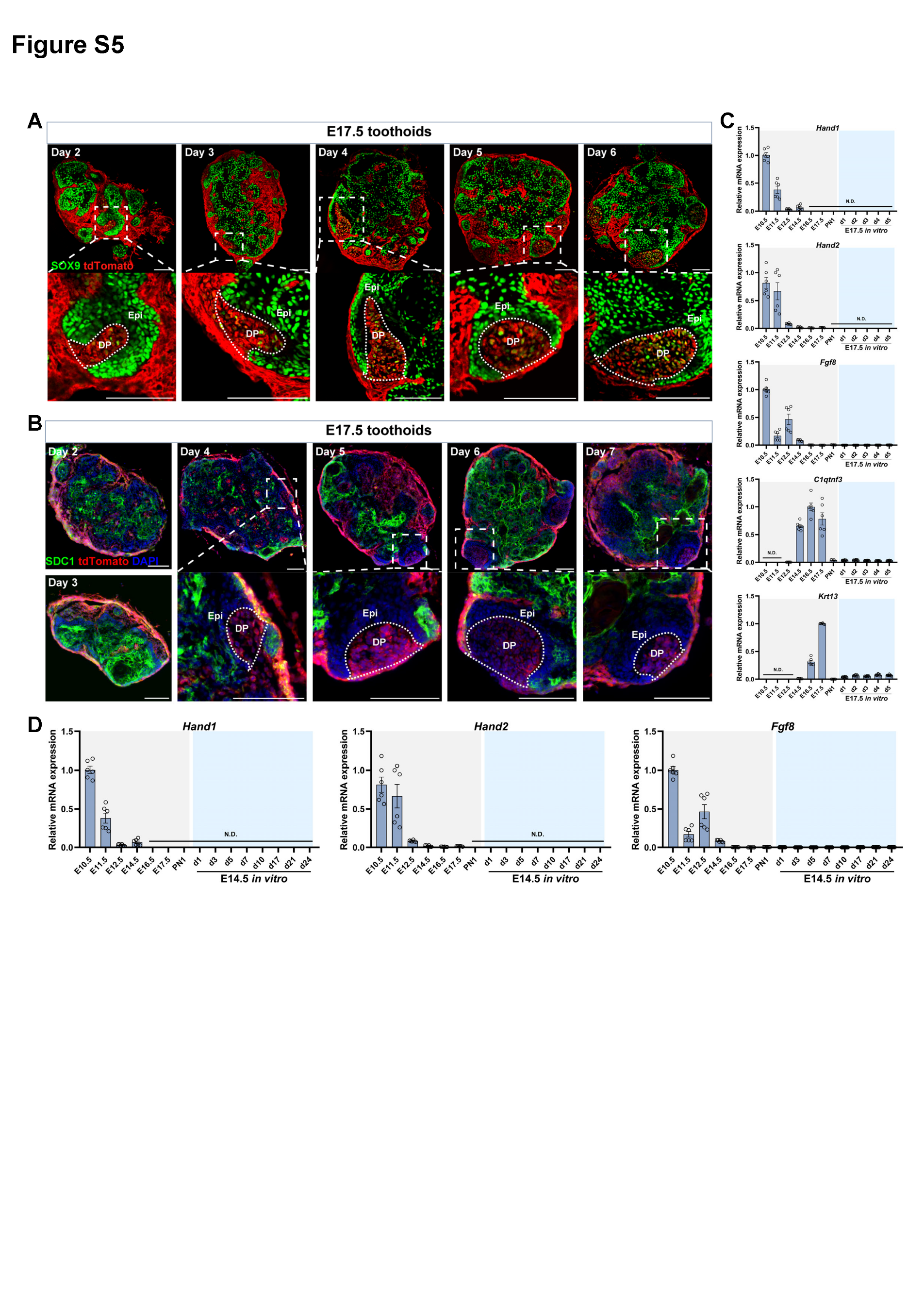

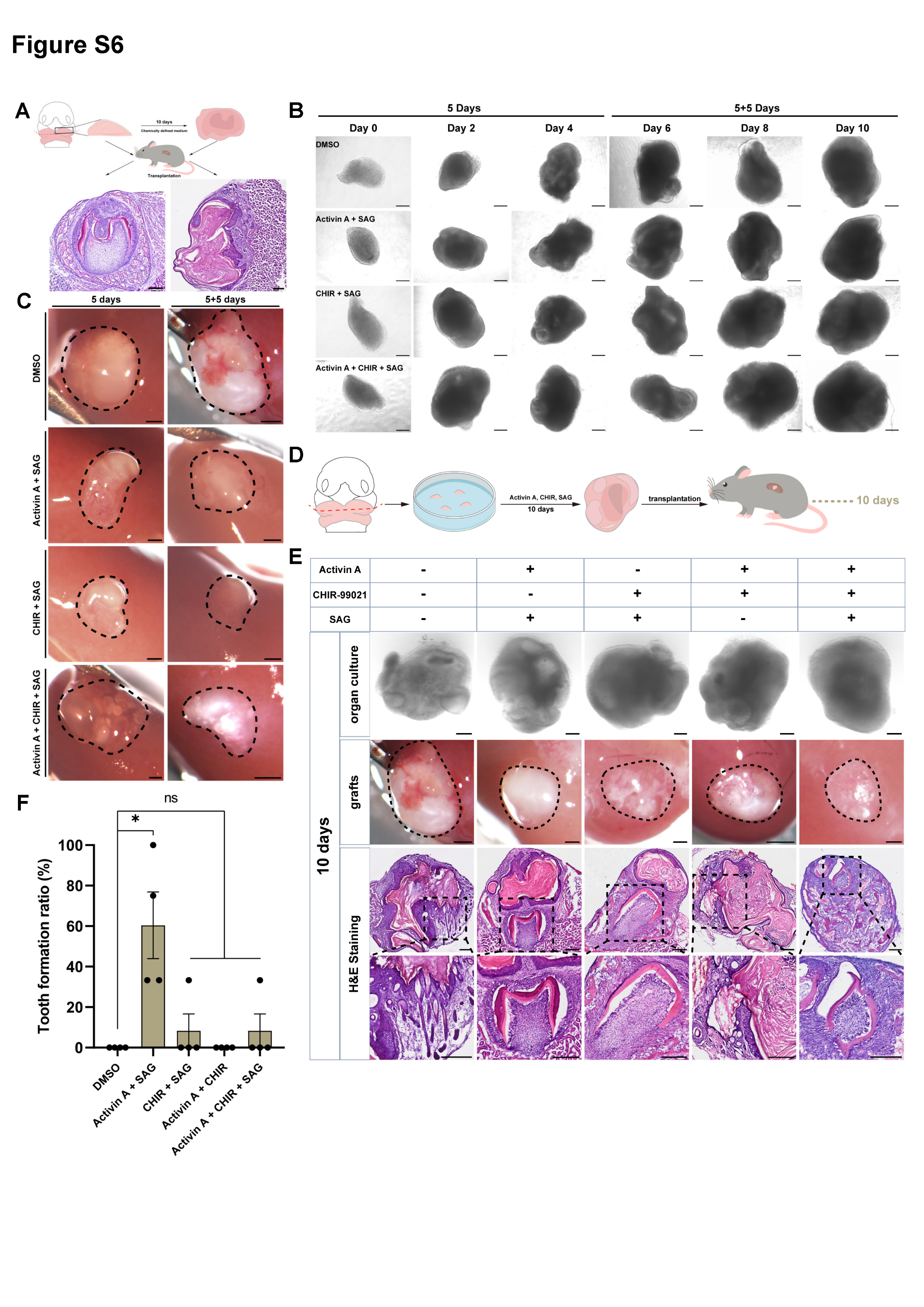

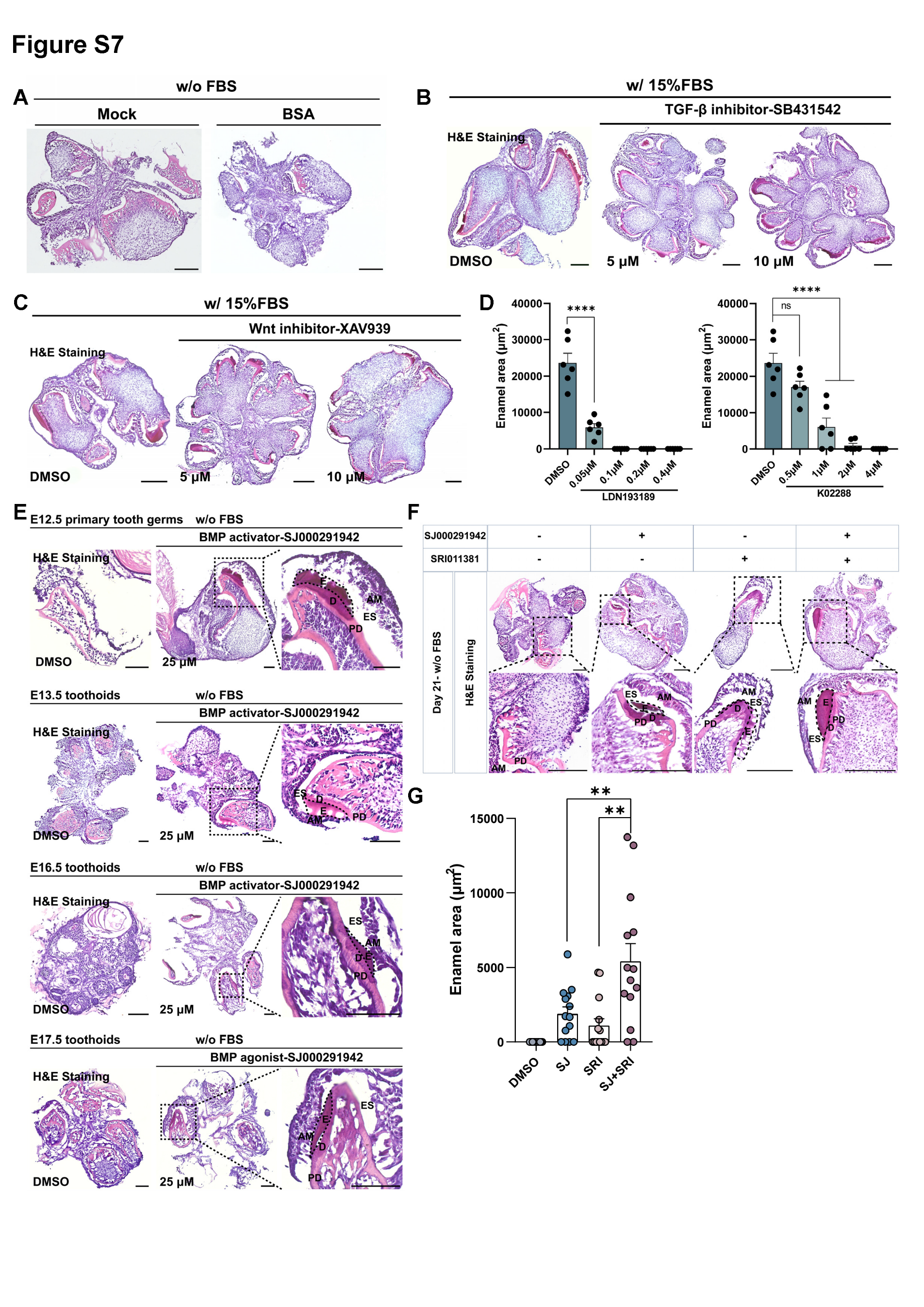

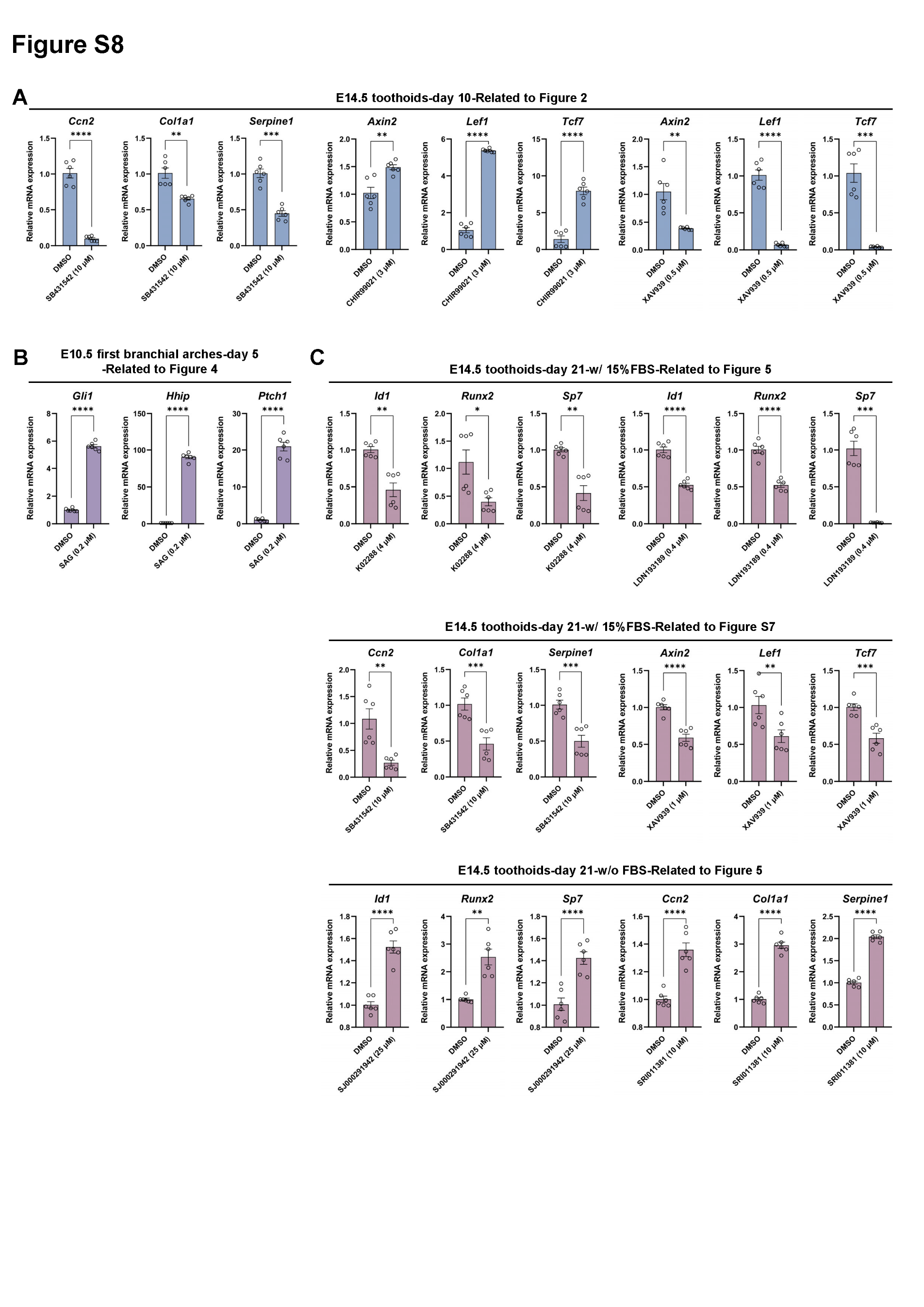


**Supplemental Tables**

**Table S1: Medium compositions.**

1. **FBS-containing toothoid culture medium**

| Component | Source | Cat. No. | Stock concentration | Final concentration | Valume (50 mL) |
| --- | --- | --- | --- | --- | --- |
| DMEM | Gibco | C11995500BT | - | - | 40.95 mL |
| FBS | Hyclone | SH30084.03 | - | 15% (v/v) | 7.5 mL |
| NEAA | Gibco | 11140050 | 100× | 1× | 500 μL |
| Pen-Strep | Gibco | 15140122 | 100× | 1× | 500 μL |
| Glutamax | Gibco | 35050061 | 100× | 1× | 500 μL |
| 2-Mercaptoethanol | Sigma | M3148 | 100 mM | 0.1mM | 50 μL |

1. **Chemically defined toothoid culture medium**

| Component | Source | Cat. No. | Stock concentration | Final concentration | Valume (50 mL) |
| --- | --- | --- | --- | --- | --- |
| GMEM | Gibco | C11710035 | - | - | 46.7 mL |
| Knockout Serum Replacement | Gibco | 10828028 | - | 1.5% (v/v) | 750 μL |
| NEAA | Gibco | 11140050 | 100× | 1× | 500 μL |
| Pen-Strep | Gibco | 15140122 | 100× | 1× | 500 μL |
| Sodium Pyruvate | Gibco | 11360070 | 100× | 1× | 500 μL |
| 2-Mercaptoethanol | Sigma | M3148 | 100 mM | 0.1mM | 50 μL |
| Matrigel | Corning | 354230 | - | 2% (v/v) | 1 mL |

**Table S2: Cell numbers and ratios for tooth reconstitution**

1. **Molar toothoids**

| Dvelopmental stage | Total cell number per toothoid (×10^4^ cells) | Ratio of epithelial to mesenchymal cells |
| --- | --- | --- |
| E13.5 | 7 | 1:4 |
| E14.5 | 10 | 1:2 |
| E16.5 | 14 | 1:2 |
| E17.5 | 20 | 1:2 |
| E18.5 | 28 | 1:1.7 |
| PN1 | 32 | 1:1.3 |

1. **Incisor toothoids**

| Dvelopmental stage | Total cell number per toothoid (×10^4^ cells) | Ratio of epithelial to mesenchymal cells |
| --- | --- | --- |
| E13.5 | 4 | 1:2 |
| E14.5 | 6 | 1:1.5 |
| E16.5 | 12 | 1:1.2 |
| E17.5 | 15 | 1:1.2 |
| E18.5 | 20 | 1:0.8 |
| PN1 | 22 | 1:0.6 |

**Table S3: Key resources table.**

1. **Antibodies used in this study**

| Antibodies | Source | Identifier |
| --- | --- | --- |
| Rabbit polyclonal anti-TurboGFP | ThermoFisher | Cat# PA5-22688; RRID: AB_2540616 |
| Goat polyclonal anti-tdTomato | SICGEN | Cat# AB8181; RRID: AB_2722750 |
| Rabbit polyclonal anti-Nestin | Huabio | Cat# R1510-19 |
| Rabbit recombinant monoclonal anti-Sp7 | Abcam | Cat# ab209484; RRID: AB_2892207 |
| Rabbit polyclonal Anti-AMGN | Santa Cruz Biotechnology | Cat# sc-32892; RRID: AB_2226455 |
| Rabbit polyclonal anti-AMBN | Santa Cruz Biotechnology | Cat# sc-50534; RRID: AB_2226393 |
| Rabbit monoclonal anti-Cd34 | Huabio | Cat# ET1606-11; RRID: AB_2924309 |
| Rabbit monoclonal anti-Postn | Abcam | Cat# ab215199; RRID: AB_2924310 |
| Rabbit monoclonal anti-Sox9 | Huabio | Cat# ET1611-56; RRID: AB_2924312 |
| Chicken polyclonal anti-Krt14 | Biolegend | Cat# 906004; RRID: AB_2616962 |
| Rabbit monoclonal anti-Lef1 | Abcam | Cat# ab137872; RRID: AB_2892647 |
| APC anti-mouse CD138 | Biolegend | Cat# 142505; RRID: AB_10960141 |
| Rabbit IgG | Abcam | Cat# ab172730; RRID: AB_2687931 |
| Goat IgG | ThermoFisher | Cat# 31245; RRID: AB_10959406 |
| Alexa ﬂuor-647 rat IgG2a, κ isotype ctrl antibody | Biolegend | Cat# 400526; RRID: AB_2864284 |
| Alexa ﬂuor-568 donkey anti goat | ThermoFisher | Cat# A11057; RRID: AB_142581 |
| Alexa ﬂuor-488 donkey anti rabbit | ThermoFisher | Cat# A21206; RRID: AB_2535792 |
| Alexa ﬂuor-647 donkey anti rabbit | ThermoFisher | Cat# A31573; RRID: AB_2536183 |

1. **Critical commercial assays**

| Name | Source | Identifier |
| --- | --- | --- |
| RNAscope Probe-Mm-Dspp | ACDbio | Cat# 448301 |
| RNAscope Probe-Mm-Fgf4 | ACDbio | Cat# 514311 |
| Opal 690 Regent | Asbio | Cat# ASOP690 |

**Table S4: Primers used in this study**

| Gene name | Primer name | Sequence (5’ to 3’) |
| --- | --- | --- |
| *Pitx2* | forward  reverse | CCTTACGGAAGCCCGAGT  AAAGCCATTCTTGCACAGC |
| *Msx1* | forward  reverse | TGCACCCTACGCAAGCACAA  GCGCTCGGCAATAGACAGGT |
| *Gapdh* | forward  reverse | GCACAGTCAAGGCCGAGAAT  GCCTTCTCCATGGTGGTGAA |
| *Hand1* | forward  reverse | AAGATCAAGACTCTGCGCCT  CAGGAGGGAAGCTTTCGGG |
| *Hand2* | forward  reverse | CCGACACCAAACTCTCCAA  GATCCATGAGGTAGGCGATG |
| *Fgf8* | forward  reverse | AACAAGCGCATCAACGCCAT  CTTGCCTTTGCCGTTGCTCT |
| *C1qtnf3* | forward  reverse | TTGTCGATTTGCCGATTTG  TCCTCCAGCTTGTGGAGACT |
| *Krt13* | forward  reverse | GGCAACGAGAAGATCACCAT  GCAAGGTCAGCTCATTTTCGT |
| *Dmp1* | forward  reverse | CGCTGAGGTTTTGACCTTGTG  GACTCACTGTTCGTGGGTGG |
| *Amelx* | forward  reverse | AGACCAAGCGGGAAGAAGTG  AGGCACAAATCATTGTGTTCTTG |
| *Ambn* | forward  reverse | GTGCCGGCATTTCCTCAAC  TGCAAGCTTCCCAACTGTCT |
| *Enam* | forward  reverse | CGACTTCTCCTCCAAAGCCA  AGCAGAGACACCAAGCAGAC |
| *Mmp20* | forward  reverse | GCTAACTACCGCCTCTTCCC  ACCTCAGTGGGACTCATGGA |
| *Runx2* | forward  reverse | CCTGAACTCTGCACCAAGTC  GAGGTGGCAGTGTCATCATC |
| *Lama3* | forward  reverse | AGCCTACTGGTCACCCTGGA  CAGGCCTGAGGTGTCGCTTA |
| *Hhip* | forward  reverse | CCCATCGGCTCTTCATTCTA  CCTTTCGTCTCCTCCCTTTA |
| *Gli1* | forward  reverse | CCAAGCCAACTTTATGTC  AGCCCGCTTCTTTGTTAA |
| *Ptch1* | forward  reverse | AAAGAACTGCGGCAAGTT  CTTCTCCTATCTTCTGAC |
| *Axin2* | forward  reverse | GGGGGAAAACACAGCTTACA  ACTGGGTCGCTTCTCTTGAA |
| *Lef1* | forward  reverse | AGGGCGACTTAGCCGACATC  CTGACCACCTCATGCCCGTT |
| *Tcf7* | forward  reverse | GACGAAGGCGAGGAACAGGA  GCGCCTTCGGACTCATTCAC |
| *Id1* | forward  reverse | GGCGAGGTGGTACTTGGTCT  CAGCTCCTTGAGGCGTGAGT |
| *Sp7* | forward  reverse | GGCCTTTCGTCTGCAACTGG  GGTGCGCTGATGTTTGCTCA |
| *Serpine1* | forward  reverse | TCCACAAGTCTGATGGCAGC  GGGGTGGTGAACTCAGTGTA |
| *Col1a1* | forward  reverse | CTGGCCCTCCTGGCAAGAAT  GAAGCCTCGGTGTCCCTTCA |
| *Ccn2* | forward  reverse | CCCTAGCTGCCTACCGACTG  AGAACAGGCGCTCCACTCTG |
